# Morphology‐Defined Entropy Streams for Random‐Bit Generation and Probabilistic Sampling in Memristor Arrays

**DOI:** 10.1002/advs.78052

**Published:** 2026-10-08

**Authors:** Hyunjun Kim, Da Ye Kim, Noah Jang, Yongho Lee, Byunghun Song, June Soo Kim

**Affiliations:** ^1^ School of Electronic and Electrical Engineering Kyungpook National University Daegu Republic of Korea; ^2^ Smart Manufacturing Innovation Center Korea Electronics Technology Institute Seongnam Republic of Korea; ^3^ Department of Electronic Engineering Cheongju University Cheongju Republic of Korea

**Keywords:** memristors, nanoislands, physical entropy, stochastic electronics, true random number generators

## Abstract

Physical entropy sources are essential for hardware security and probabilistic information processing, yet most memristor‐based random‐number generators rely on stochastic switching outputs without identifying a designable materials‐level parameter that controls how intrinsic device stochasticity is expressed. Here, we present a morphology‐engineered entropy platform based on Ag/TiO_2_/Au nanoisland (NI)/Pt electrochemical memristor arrays, in which the Au NI morphology acts as a tunable structural modulator of the stochastic high‐resistance‐state transport landscape. Thickness‐controlled Volmer–Weber growth produces distinct NI regimes that reshape local‐field hotspots and candidate filament pathways through which residual filament configurations, interfacial defects, and stochastic ion migration are translated into HRS‐current dispersion. NI‐1.5 provides a practical entropy‐engineering window where broad hotspot diversity and recoverable HRS states are balanced. The morphology‐modulated HRS dispersion is converted into entropy streams in a 32 × 32 array through paired‐current comparison and validated using bit probability, binary entropy, min‐entropy, restart/reproducibility tests, autocorrelation, NIST SP 800–22 tests, and machine‐learning‐based predictability evaluation. The validated stream further serves as a stochastic sampling primitive for Monte Carlo probabilistic inference. This work establishes nanoscale morphology as a designable materials‐level control parameter linking stochastic memristive transport, array‐level randomness, and probabilistic sampling.

## Graphical abstract

Au nanoisland morphology defines stochastic high‐resistance‐state transport in Ag/TiO_2_ memristor arrays, transforming nanoscale structural disorder into a tunable physical entropy resource. Morphology‐controlled field hotspots and reset‐modulated nanogaps generate statistically validated random‐bit streams, which further serve as device‐derived stochastic sampling primitives for Monte Carlo probabilistic inference.

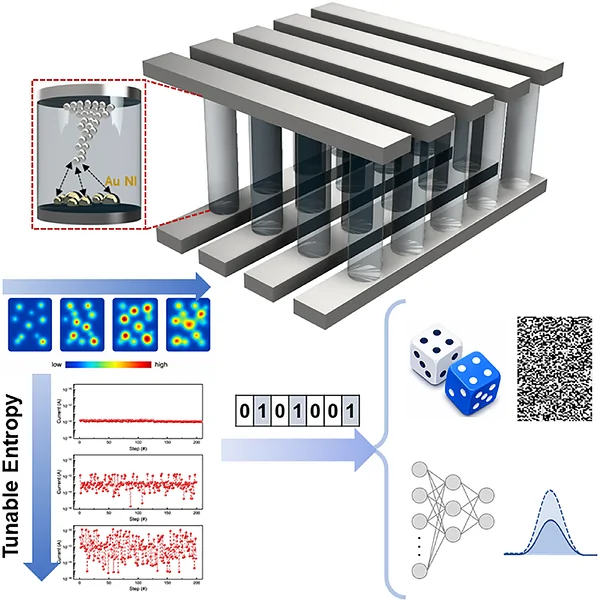

## 1 Introduction

True random number generators (TRNGs) are essential for secure electronics, identity authentication, and stochastic information processing because their outputs originate from physical entropy rather than deterministic algorithms [1, 2]. As edge computing, cyber‐physical systems, and AI‐enabled hardware continue to expand, there is growing demand for TRNG platforms that are compact, energy efficient, and physically auditable at the device level [1, 3, 4]. Beyond conventional security functions, physically unpredictable randomness is also increasingly relevant to entropy‐sensitive computational frameworks, including stochastic sampling in generative models and related inference schemes [5, 6, 7, 8, 9]. These trends motivate hardware platforms that can not only produce statistically validated randomness, but also reveal and control its physical origin.

Existing hardware TRNG approaches, including ring‐oscillator jitter, noise‐coupled analog‐to‐digital converters, magnetic tunnel junctions, spintronic devices, and spin‐crossover systems, provide valuable routes toward physical randomness generation [10, 11, 12, 13, 14]. However, many of these approaches rely on circuit‐level complexity, external peripheral components, or nontrivial power overhead, motivating the search for simpler nanoscale devices in which entropy generation is directly embedded in material response and switching physics. Memristors are therefore attractive candidates because they combine compact form factors, dense crossbar compatibility, and intrinsic stochasticity arising from nanoscale ionic and electronic processes [15, 16, 17].

Despite this promise, most memristor‐based TRNG studies still validate randomness primarily at the output level by exploiting stochastic observables such as switching‐delay variation or random telegraph noise, while stopping short of identifying a structurally designable entropy source that can be quantitatively linked to nanoscale device geometry [15, 16, 18]. As a result, randomness is often treated as an empirical byproduct of filamentary switching rather than as a physically engineered device function. This limitation is important because the quality, predictability, and long‐term controllability of random numbers are ultimately shaped by nanoscale features that govern local field concentration and transport pathways, including morphology, curvature, and interfacial gap geometry [17, 18]. Without quantitatively establishing such structure‐to‐entropy relationships, it remains difficult to rationally design memristor TRNGs whose entropy can be tuned, optimized, and physically audited for practical hardware systems. In this context, structural disorder should be regarded not merely as a reliability penalty to be minimized, but as a functional nanoscale resource that can be engineered for entropy generation.

A central unresolved question is therefore how nanoscale morphology defines the stochastic switching landscape in filamentary memristors. When electric‐field localization is governed by heterogeneous surface topography, local curvature, and interfacial spacing, conductive‐filament evolution can become strongly probabilistic in a manner that is not merely noisy, but structurally encoded. Establishing this morphology‐to‐entropy relationship would move the field beyond output‐level demonstration of NIST‐pass behavior and toward a device‐physics framework for controllable entropy engineering. Such a framework is especially relevant for applications that require both statistical randomness and a traceable physical origin, including hardware security and entropy‐aware computational systems [1, 17, 19].

In this work, we present a morphology‐engineering strategy for controlling HRS stochastic transport in Ag/TiO_2_/Au nanoisland (NI)/Pt electrochemical memristor arrays and converting it into a physical entropy stream. The Au NI layer forms thickness‐dependent island density, size distribution, and coalescence states through Volmer–Weber growth, thereby modulating local electric‐field hotspots, filament/nanogap formation pathways, and reset‐induced HRS‐current dispersion. Here, the stochastic HRS fluctuation is not treated merely as device variability, but as a morphology‐defined entropy resource that can be converted into information functionality. The extracted entropy stream is transformed into random bitstreams by paired‐current comparison in a 32 × 32 array and statistically validated using entropy, bit uniformity, autocorrelation, NIST SP 800–22 tests, and machine‐learning‐based predictability analysis. Beyond random‐bit generation, we further evaluate whether the validated entropy stream can serve as a stochastic sampling source for Monte Carlo probabilistic inference. This work therefore links Au NI morphology, stochastic transport, array‐level entropy validation, and probabilistic sampling fidelity within a unified materials‐to‐information‐function framework.

## 2 Results and Discussion

### 2.1 Overview of Morphology‐Defined Entropy Engineering in the Au NI Memristor Array

Figure 1 summarizes the overall concept of this work. In the Ag/TiO_2_/Au NI/Pt memristor array, the Au NI interlayer acts as a structural parameter that modulates the stochastic transport landscape. Owing to Volmer–Weber growth, Au forms thickness‐dependent island morphologies ranging from sparse and distributed islands to partially and extensively coalesced structures. This morphology evolution modifies the statistical distribution of candidate filamentary pathways and interfacial transport configurations, thereby modulating the width, reproducibility, and recoverability of the HRS‐current distribution.

**FIGURE 1:**
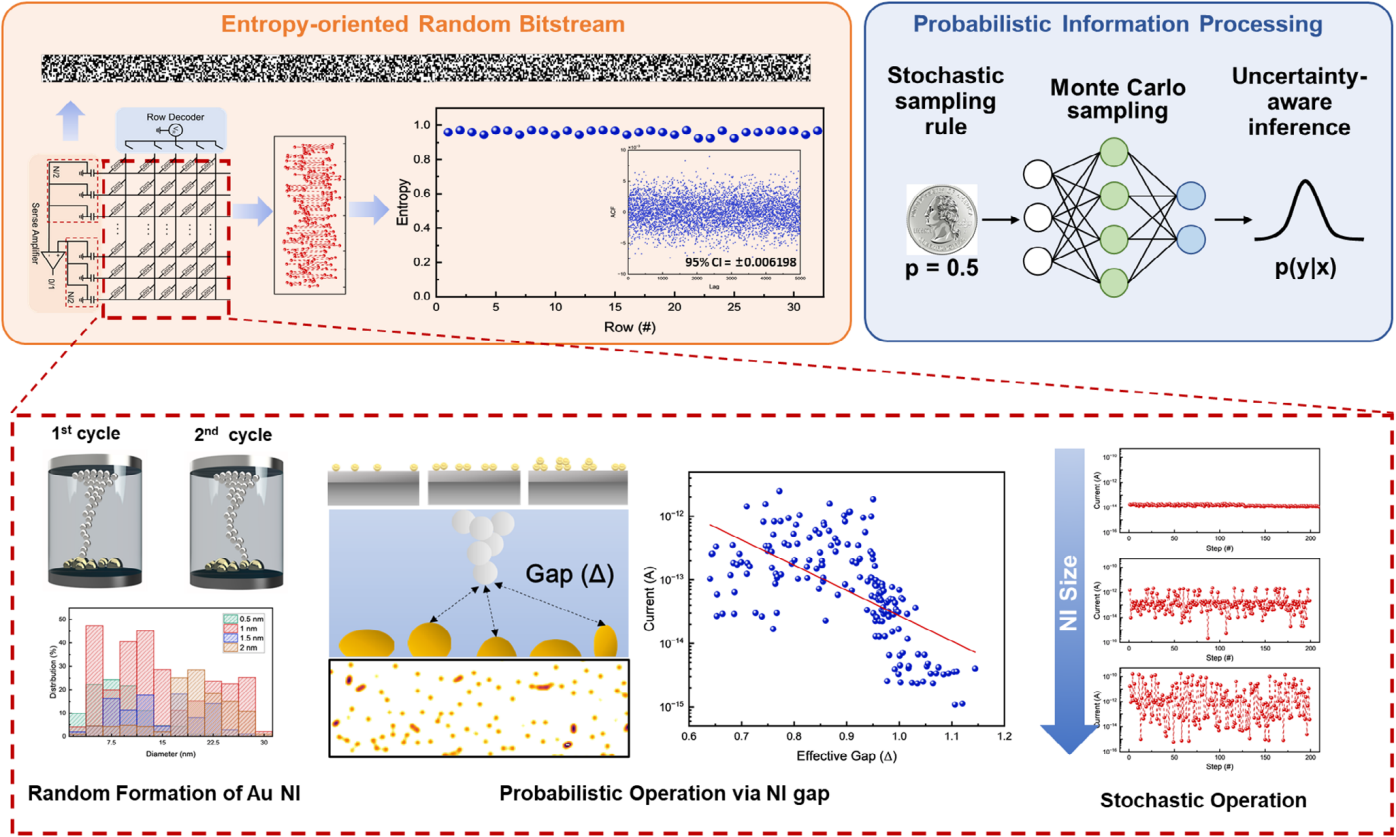
Overview of morphology‐engineered entropy generation in the Ag/TiO_2_/Au NI/Pt memristor platform. Au NI morphology provides a tunable structural landscape with spatially heterogeneous field concentration and multiple candidate filament pathways. Together with cycle‐dependent residual‐filament, defect, ionic, and interfacial transport states, this landscape produces stochastic HRS‐current distributions that are digitized into random bitstreams in a 32 × 32 crossbar array.

A central feature of this study is that such HRS fluctuation is not interpreted simply as undesirable instability. The entropy stream examined here arises from the coupled action of morphology‐related field heterogeneity and cycle‐dependent microscopic factors, including residual Ag‐filament configurations, interfacial defects, trap occupation, and stochastic ion migration. The Au NI morphology is therefore treated as a tunable structural parameter that controls the statistical expression of these intrinsic ECM fluctuations, rather than as their sole microscopic origin. In underdeveloped NI morphologies, hotspot diversity is insufficiently formed, resulting in a narrow HRS distribution and limited entropy. In contrast, over‐coalesced morphologies promote dominant conduction paths and poor HRS recovery. The NI‐1.5 condition provides a balanced regime in which broad hotspot diversity and recoverable HRS states coexist, defining an entropy/sampling window where morphology‐modulated stochasticity and device operability are simultaneously satisfied. The extracted stream is then statistically validated and connected to Monte Carlo stochastic sampling. Therefore, Figure 1 frames this work as a morphology‐engineering approach for controlling the statistical landscape of a multivariable physical entropy source.

### 2.2 Origin of Probabilistic Switching in the Memristor

This work utilizes VW‐grown Au NIs as structural modulators of probabilistic switching and HRS entropy generation in ECM memristors. The Ag/TiO_2_/Au NI/Pt metal–insulator–metal structure integrated into a crossbar array is shown in Figure 2A. The cross‐sectional FE‐TEM image (Figure 2B) confirms the stacked layers, and the Au NI region appears as a non‐uniform, discontinuous layer where Ag filaments repeatedly form and rupture between the top and bottom electrodes. The top‐view SEM image of a 1.5 nm NI sample (NI 1.5) shows a discontinuous film composed of aggregated Au structures, with island diameters ranging from 5–30 nm and heights from 1–10 nm (Figure 2C,D). To further analyze the growth process, NI samples with thicknesses from 0.5 to 2 nm were prepared (Figure S2). As shown in Figure S2, the 1.5 nm deposition condition produced the widest distribution of island diameters and heights. Grazing‐incidence x‐ray diffraction (GIXRD) was performed to examine structural evolution during NI growth. The results indicate that the deposited Au exists as nanocrystalline islands with preferential (111) orientation on Pt (Figure 2E) [20, 21, 22, 23]. The 2 nm NI sample exhibited a larger crystalline domain, and its scattering intensity increased by ∼2.8×, indicating significant nucleation and crystallization progression. The 2θ peak for the Au (111) plane shifted to a higher angle compared with bulk Au (≈38.24°) [24, 25], indicating a reduced lattice constant. This shift is consistent with lattice compression caused by epitaxial interaction with the underlying Pt substrate, which has a smaller lattice parameter (a_Pt_ ≈ 3.923 Å < a_Au_ ≈ 4.078 Å) [26, 27, 28, 29]. Quantitative XPS analysis of a companion titanium‐oxide film deposited under the same electron‐beam evaporation conditions yielded an RSF‐corrected O/Ti atomic ratio of 2.23. Together with the Ti4^+^‐dominated Ti 2p spectrum and the principal lattice Ti–O component in the O 1s spectrum, this result supports a near‐stoichiometric TiO_2_‐like composition (Figure S2).

**FIGURE 2:**
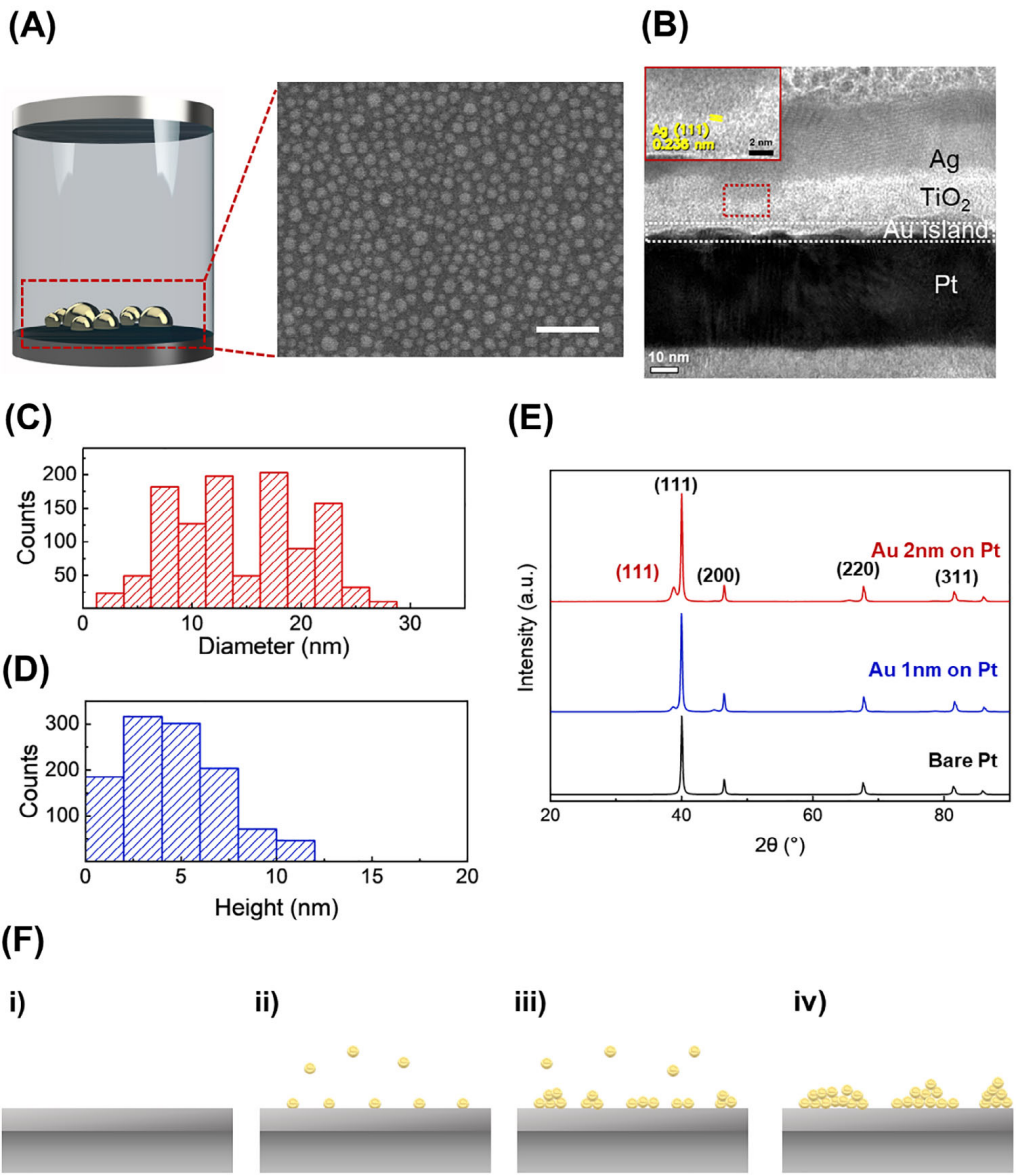
(A) Structure of the TiO_2_/Au NI–based memristor and SEM image of the Au NI. (B) Cross‐sectional HR‐TEM image of the memristor stack. (C) Diameter and (D) height distributions of the NIs, showing variations in their physical characteristics. (E) GIXRD results of Au NI with different deposited thicknesses. The Au diffraction peaks are marked in red, whereas the Pt substrate peaks are marked in black. (F) Schematic illustration of Au NI growth via the VW mechanism.

These observations confirm that the Au NIs form through the VW growth mechanism (Figure 2F) [30, 31, 32, 33]. During early deposition, Au atoms arriving at the Pt surface adsorb as adatoms. Because the spreading parameter S = γ_s_ − (γ_f_ + γ_i_) is negative under non‐wetting conditions, Au atoms favor cohesion with one another rather than forming a wetting layer [32, 34]. The surface diffusion length *l_D_*, limited to a few nanometers at the low deposition rate of 0.1 Å/s, promotes the formation of three‐dimensional nuclei at multiple sites, a hallmark of VW‐type island growth [35, 36, 37]. The NI size distribution is strongly influenced by deposition thickness (Figure S2). At 0.5–1.5 nm, dense nucleation yields a high island density, but individual islands remain small due to limited growth, producing a uniform distribution of small NIs. At 2 nm, coalescence and early‐stage dewetting occur, causing island aggregation and growth into larger structures while reducing overall geometric diversity despite increasing the average size. The 1.5 nm condition represents a transitional Goldilocks regime. At this thickness, three processes, i.e., new nucleation (small NIs), accelerated growth (medium NIs), and aggregation (large NIs), occur simultaneously. This arises because the surface diffusion length becomes comparable to the average NI spacing, producing islands with different growth histories. As a result, the NI‐1.5 sample exhibits the broadest and most asymmetric geometric distribution, corresponding to the maximum achievable shape diversity and thus highest entropy potential.

### 2.3 Probabilistic Switching of Memristors by Au NI

The DC *I*–*V* characteristics of the developed memristor over 200 cycles are shown in Figure 3A. The device exhibits two distinct states: a low‐resistance state (LRS) and a high‐resistance state (HRS), corresponding to the formation and rupture of Ag filaments, respectively. This switching behavior is characteristic of ECM devices, and a positive temperature coefficient of resistance (TCR) is observed in the LRS [38, 39] (Figure S3). The device demonstrates a memory window exceeding 10^6^ during cyclic measurements without an electro‐forming step, along with high endurance over 10^5^ cycles and stable retention over 10^6^ s at a broad temperature range (Figures S4 and S5). In the present TRNG operation, retention is not the physical origin of randomness; rather, it ensures that the stochastic HRS established after each reset operation remains sufficiently stable during the subsequent read and paired‐current comparison. Because the measured retention time greatly exceeds the reset–read–comparison time scale, it provides a sufficient stability margin for reliable bit extraction. The randomness quality is independently validated through entropy, min‐entropy, bit‐uniformity, autocorrelation, restart and reproducibility tests, machine‐learning‐based predictability analysis, and NIST SP 800–22 tests. Noticeable cycle‐to‐cycle variations appear in the *I*–*V* curves. The HRS current distribution is considerably broader than that of the LRS, and the set voltage (V_SET_) exhibits significant fluctuation. The distributions of the HRS current at 0.2 V and V_SET_ across cycles are presented in Figure 3B,C, respectively. V_SET_ varies between 2.5 and 4.5 V randomly, while the HRS current fluctuates by more than five orders of magnitude. This broad stochasticity in V_SET_ and HRS current forms the experimental basis of the present entropy‐engineering platform and motivates the following analysis of its structural origin at the Au NI interface (Figure 3D).

**FIGURE 3:**
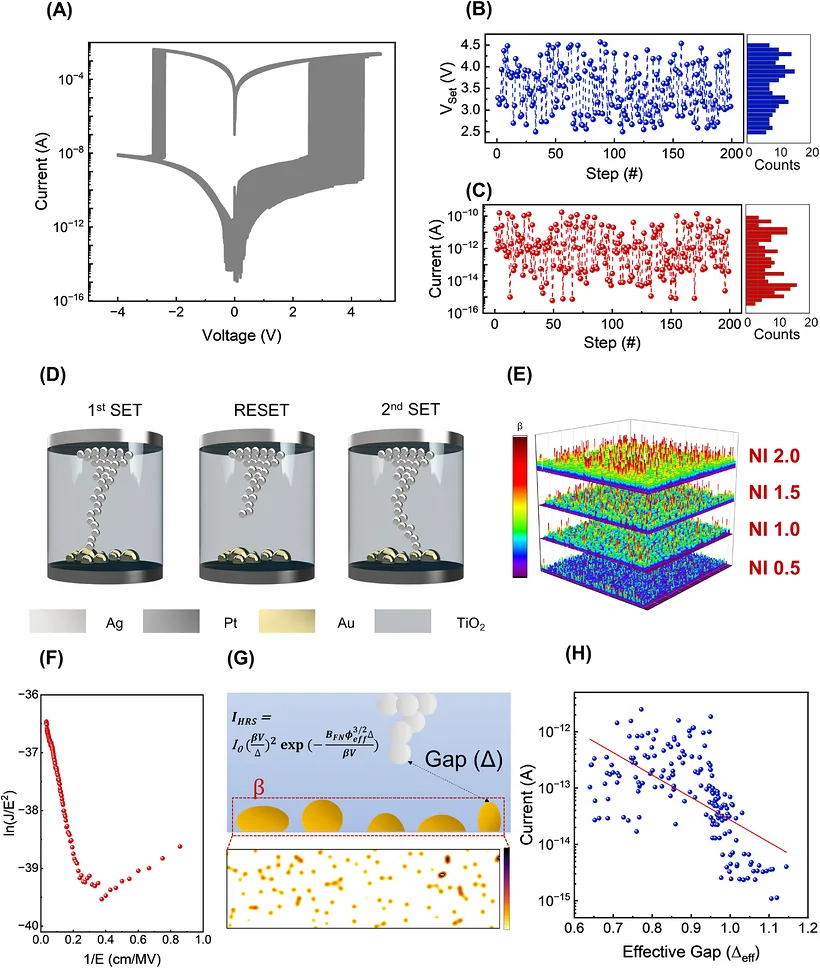
(A) *I*–*V* characteristics of the memristor. (B) V_SET_ distribution over 200 cycles. (C) HRS current distribution measured at 0.2 V. (D) Schematic illustration of stochastic switching modulated by Au‐NI morphology. (E) β‐mapping of Au‐NI under different deposition conditions. (F) Representative FN linearization of the common pre‐SET HRS regime. (G) Schematic of morphology‐modulated probabilistic HRS operation, illustrating morphology‐modulated variation in the effective interfacial transport state. (H) Relationship between the fitted effective transport‐gap parameter Δ_eff_ and HRS current within the adopted FN‐type framework.

In ECM devices, V_SET_ behaves as an extreme‐value variable determined by the location at which critical filament nucleation first occurs among multiple candidate sites, represented here by the individual Au NI. The synthesized NIs exhibit geometries with pronounced curvature at ridges and vertices, which amplify the local electric field (E_local_) according to:

(1) $$E_{local}=\beta_{i}V/t$$

Where 𝛽_𝑖_ is the shape‐dependent field enhancement factor, and t is the effective insulator thickness [40, 41, 42]. The increased local field exponentially lowers the activation barrier for Ag^+^ migration and reduction, making the delay time for filament nucleation at each candidate site (τ_𝑖_) highly sensitive to local field variations and approximately following [43, 44, 45]:

(2) $$\tau_{i}\left(V\right)∼\tau_{0}\exp\left(A/E_{local}\right)$$

where τ_0_ is the attempt time and A is the field‐activation parameter. During voltage ramping, variations in β_i_, residual filament segments after the reset process, and microscopic defects broaden the distribution of delay times exponentially, resulting in the observed V_SET_ fluctuations between cycles [46, 47, 48, 49]. The NI configuration used in this work lies in a morphological transition regime, where numerous hot spots with comparable geometric advantages coexist due to the island density and spacing, further reinforcing the probabilistic switching behavior [50, 51]. Cycle‐to‐cycle stochasticity in ECM memristors originates from the coupled evolution of multiple microscopic variables, including residual Ag‐filament configurations, local defect and trap distributions, stochastic ion migration and reduction, and interfacial inhomogeneity. Au NI morphology should therefore not be interpreted as the sole microscopic origin of individual switching events. Rather, it provides a persistent and tunable structural boundary condition that modulates the statistical availability of candidate filament nucleation, rupture, and recovery pathways. Previous studies have shown that metal nanoparticles or nano‐islands can improve switching uniformity when local field enhancement confines conductive‐filament formation to a limited number of reproducible preferential sites [52, 53]. The statistical consequence of such field localization, however, depends on the morphology and distribution of the field‐enhanced sites as well as on the switching state being considered. In the present device, the heterogeneous NI morphology provides a broad distribution of field‐favored candidate pathways rather than a single dominant confinement site. Moreover, the entropy source examined here is the post‐RESET HRS current, which reflects cycle‐dependent filament dissolution, residual Ag configurations, interfacial transport states, trap occupation, and ionic redistribution in addition to filament nucleation. Accordingly, dominant preferential sites can promote uniform switching, whereas multiple competing field‐favored pathways combined with reset‐dependent residual states can broaden the accessible HRS distribution.

This interpretation is supported by the systematic and non‐monotonic evolution observed across the complete NI‐thickness series under otherwise identical fabrication and measurement conditions. NI‐0.5 exhibits a relatively narrow and stable HRS distribution, NI‐1.0 shows moderate broadening, NI‐1.5 provides the broadest reproducible HRS dispersion while maintaining reliable HRS recovery, and the over‐coalesced NI‐2.0 condition exhibits degraded recovery and frequent low‐resistance failure. Because residual‐filament fluctuations, defects, traps, and stochastic ionic motion are intrinsic to all NI conditions, this reproducible morphology dependence supports Au NI morphology as a major controllable structural modulator of the switching statistics rather than as their exclusive microscopic origin.

The AFM‐derived local‐gap‐proxy and curvature analyses provide comparative morphology‐informed descriptors of geometrically prominent regions but do not directly measure the local electric field or filament position. The reset‐amplitude dependence further identifies the residual‐filament and interfacial transport states as dynamic contributors, while the multi‐device measurements confirm that the broad stochastic switching behavior is reproducible across independent cells. Accordingly, the present results establish a systematic morphology–electrical correlation and a physically plausible intermediate mechanism, but not a one‐to‐one causal correspondence between an individual NI and a specific switching event.

The morphology and β‐mapping results for different NI conditions (Figure 3E and Figure S7) indicate distinct regimes. At NI‐0.5, dense islands with the smallest average diameter and relatively low β values form; the relatively narrow β distribution is consistent with stable ECM switching during cyclic operation. As the nominal Au thickness increases (NI‐1.0 and NI‐1.5), the average island diameter increases and the β distribution broadens. This wide dispersion of diameters and β is consistent with multiple spatially distributed high‐β regions and an increased statistical availability of geometrically distinct candidate transport pathways. Under these conditions, the broader cycle‐to‐cycle variability constitutes a practical entropy source for the device. Importantly, under the present device configuration, Au NI morphology acts as a tunable structural modulator of the statistical entropy landscape, consistent with the systematic evolution of the morphology‐derived field‐concentration descriptors and HRS‐current statistics. In contrast, at NI‐2.0 the deposited Au approaches a percolated network or quasi‐continuous film rather than remaining as discrete islands.

The β‐map for NI‐2.0 shows spatially extended high‐β regions that coincide with degraded HRS recovery and persistent low‐resistance conduction. Indeed, as shown in Figure S6A, devices with NI‐2.0 fail to recover the HRS within fewer than 20 cycles. Device‐to‐device statistics were further collected across the full NI‐thickness series to confirm that the optimized NI‐1.5 behavior is not a selected‐device artifact (Figure S6B). For each condition, ten devices were evaluated, and the HRS current at 0.2 V was analyzed on a logarithmic scale. NI‐0.5 showed stable but weakly stochastic HRS behavior, whereas NI‐1.0 produced moderate broadening of the HRS distribution. NI‐1.5 yielded the largest reproducible HRS dispersion while maintaining high recovery yield, whereas NI‐2.0 exhibited degraded HRS recovery and frequent low‐resistance failure. Because residual filament fluctuations, defects, and ionic stochasticity are present in every condition, this reproducible and non‐monotonic morphology dependence supports the role of Au NI morphology as a controllable structural modulator. NI‐1.5 is therefore identified as the practical entropy‐engineering window in which morphology‐modulated stochasticity and device operability are balanced. To verify device‐to‐device reproducibility, 100‐cycle *I*–*V* measurements were performed on ten independent NI‐1.5 devices (Figure S6C). All devices exhibited forming‐free bipolar switching with a broad distribution of V_SET_ and HRS values. These results confirm that the stochastic switching behavior is reproducible across multiple cells. The device‐resolved CDFs of the HRS resistance values converted from the corresponding 0.2‐V HRS‐current measurements are shown in Figure S6D. The CDFs exhibit extended quasi‐linear central regions in log‐resistance space, supporting a broad stochastic HRS distribution.

These observations motivate a more detailed evaluation of the HRS transport mechanism. The positive‐voltage pre‐SET HRS branches extracted from the room‐temperature *I*–*V* cycles were first compared using Fowler–Nordheim (FN), Poole–Frenkel (PF), Schottky, field‐assisted trap/tunneling, and power‐law transport models over an identical 0.30–2.40 V interval, which lies below the minimum measured switching onset of approximately 2.57 V (Figure 3F) [54, 55, 56, 57, 58]. Each fitted model was reconstructed in the common ln(I) domain and evaluated using the root‐mean‐square error and Akaike information criterion. The FN‐type formulation yielded the lowest AIC in approximately 96.2% of the analyzed cycles among the four field‐assisted mechanisms and remained preferred in approximately 85.0% after inclusion of the power‐law control. The median ln(I)‐domain RMSE values were 0.0907, 0.2561, 0.3277, 0.2932, and 0.1467 for the FN, PF, Schottky, field‐assisted trap/tunneling, and power‐law models, respectively. Fitting‐window sensitivity analysis further yielded FN‐selection ratios of approximately 81%–88%. These cycle‐level comparisons identify the FN‐type formulation as the most consistent comparative description of the bounded room‐temperature pre‐SET HRS regime among the tested transport models.

Temperature‐dependent HRS *I*–*V* measurements from 25°C to 85°C were then performed to experimentally examine whether the high‐field FN‐type scaling is retained across temperature (Figure S11). The apparent activation energy decreases from approximately 0.09–0.14 eV at representative voltages of 0.5–0.8 V to approximately 0.03–0.035 eV in the 1.2–2.0 V region. Consistently, voltage‐based FN representations, ln(I/V^2^) vs. 1/V, evaluated over the common 1.0–2.0 V high‐field interval, retain negative slopes at all measured temperatures, with R^2^ values of 0.982, 0.993, 0.974, and 0.983 at 25°C, 45°C, 65°C, and 85°C, respectively. Together, the room‐temperature cycle‐level model comparison and the temperature‐dependent FN analysis support a field‐dependent mixed‐transport picture in which thermally activated trap‐ and interface‐related contributions are more pronounced at lower voltages, whereas FN‐type field‐driven tunneling becomes increasingly prominent toward the high‐field pre‐SET HRS regime. Accordingly, the FN‐type formulation is used as a comparative transport framework for analyzing the high‐field pre‐SET HRS response.

Within this framework, the fitted slope and intercept are treated as effective transport descriptors rather than independently determined material constants. The HRS response is parameterized using the local field‐amplification factor β and an effective transport‐gap parameter Δ_eff_. Here, Δ_eff_ represents the model‐equivalent interfacial transport state required to reproduce the measured current under fixed assumptions regarding the effective barrier, local field enhancement, and barrier geometry [55, 57, 58, 59, 60]. It is therefore not interpreted as a direct geometric measurement of the physical separation between the residual Ag filament and the counter electrode. When the same assumptions are applied throughout the analyzed cycles, relative changes in Δ_eff_ provide a consistent basis for comparing reset‐dependent HRS transport states, whereas the absolute values remain model‐dependent. Within this bounded FN‐type phenomenological framework, the fitted slope and intercept provide apparent transport parameters rather than independently measured material constants. The HRS response is parameterized using the local field‐amplification factor β and an effective transport‐gap parameter Δ_eff_.

For the subsequent comparative calculations, the model‐dependent field‐related factor is denoted β_eff_ and is not numerically equated with the AFM‐derived relative β map. The local transport field can be approximated as [57, 58]:

(3) $$E≅\beta_{eff}V/\Delta_{eff}$$

Accordingly, the FN‐type current can be written as [54, 55, 57, 58]:

(4) $$J_{FN}\propto {\left(\frac{\beta_{eff}V}{\Delta_{eff}}\right)}^{2}\exp\left(-\;\frac{B_{FN}\phi_{eff}^{3/2}\Delta_{eff}}{\beta_{eff}V}\right)$$

Here, φ_eff_ is the effective barrier height. Because the fitted slope and intercept constrain coupled combinations of φ_eff_, β_eff_, transport length, effective conduction area, and barrier geometry, these quantities are not independently identifiable from the measured *I*–*V* characteristics alone. Accordingly, Δ_eff_ is used only as a model‐equivalent descriptor under common assumptions.

The extracted Δ_eff_ is a model‐equivalent transport parameter and not a direct geometric measurement of the physical filament–electrode separation. Its absolute value depends on the assumed barrier height, local field‐enhancement factor, barrier geometry, trap‐mediated contributions, and dominant conduction pathway. When identical assumptions are applied to all cycles, the relative evolution of Δ_eff_ can be used to compare reset‐dependent transport states, but it cannot uniquely separate changes in physical distance, barrier height, trap occupation, and local field concentration.

Field concentration around nanoscale protrusions depends on the complete local geometry and electrostatic boundary conditions, including protrusion height, curvature, aspect ratio, lateral shape, spacing, and neighboring‐feature screening [40, 41, 42]. For a controlled relative comparison of the AFM morphologies, β is operationally defined as a normalized relative field‐concentration proxy under a common one‐dimensional local‐separation approximation. Under the assumptions of a nominally planar counter boundary, a homogeneous dielectric, and a predominantly vertical field, the planar reference field and local field estimate are expressed as

(5) $$E_{0}=\frac{V}{G},\;E_{1D}\left(x,y\right)=\frac{V}{G-h\left(x,y\right)}$$

where G is the common nominal reference separation applied consistently to all NI conditions, and h(x,y) is the local AFM height relative to the reference plane. The normalized morphology‐derived proxy is therefore defined as

(6) $$\beta \left(x,y\right)\equiv \frac{E_{1D}\left(x,y\right)}{E_{0}}=\frac{G}{G-h\left(x,y\right)}$$

For |h/G|<1, the corresponding formal expansion is

(7) $$\beta =1+\frac{h}{G}+{\left(\frac{h}{G}\right)}^{2}+\cdots$$

The unity term therefore follows directly from normalization to the planar reference condition at h = 0, for which β = 1. The series expansion is included only to clarify this normalization; the exact expression G/[G−h(x,y)] is used to generate the comparative β maps. The same numerical treatment is applied to all NI conditions, enabling an internally consistent comparison across the complete morphology series.

The independently calculated curvature map is retained as a qualitative descriptor of summits, rims, depressions, and rapidly varying topographic regions; curvature is not used as a direct quantitative input in Equation 6. Full three‐dimensional effects, including inter‐island screening, lateral electrostatic coupling, complete island shape and aspect ratio, dielectric inhomogeneity, electrode roughness, possible topographic modification during TiO_2_ deposition, and the cycle‐dependent residual‐filament configuration, are outside the present one‐dimensional approximation [40, 41, 42]. Accordingly, the β analysis is used for relative morphology comparison and statistical morphology–electrical correlation without assigning absolute local‐field values or individual filament‐nucleation sites.

The dependence of the HRS distribution on reset level was investigated by varying the reset voltage (Figure S8). Increasing the reset amplitude lowers the median HRS current while widening its distribution. This behavior is consistent with progressive modification of the residual filament and interfacial transport state at stronger reset levels. A more strongly ruptured or weakly coupled residual conduction configuration could increase the effective transport barrier or transport length; however, the present electrical analysis does not directly resolve the physical filament geometry or rupture position. Within the FN‐type framework, Δ_eff_ was calculated for each cycle using identical assumptions (Figure 3G,H). Lower HRS currents correspond to larger fitted Δ_eff_ values, but because Δ_eff_ is mathematically inferred from the measured current, this relationship is interpreted as an internal consistency of the phenomenological model rather than independent evidence of an actual geometric nanogap. More importantly, the independently controlled reset amplitude systematically changes the HRS‐current distribution, supporting the residual filament and interfacial state as an important dynamic contributor. Cyclic responses for the NI‐thickness series further show that morphology provides the persistent structural landscape within which these reset‐dependent states evolve (Figure S8). At NI‐2.0, the fitted Δ_eff_ decreases markedly together with loss of HRS recovery and persistent low‐resistance conduction; this behavior is described as a change in the effective transport state rather than a directly observed collapse of a physical gap. Taken together, the morphology analysis, β‐mapping, comparative transport analysis, and reset‐level dependence support a multivariable mechanism in which a morphology‐modulated field landscape interacts with cycle‐dependent residual filament, defect, ionic, and interfacial transport states to generate the observed entropy distribution.

A 32 × 32 memristor crossbar array was fabricated and connected to an experimentally implemented capacitor‐integration/sense‐amplifier readout circuit for direct random‐bit generation (Figure 4A–C). Figure 4B shows the broad cumulative probability distribution of HRS currents across the array. For each selected row, the 32 columns were divided into 16 paired‐column sets. The paired HRS currents were integrated at the corresponding capacitive sense nodes, and the resulting sense‐node voltages were physically compared by the sense amplifier to directly produce binary outputs. The implemented readout circuit is shown in Figure S9A,B. Thus, analog HRS outputs were converted into digital random bits by the readout hardware rather than by offline numerical comparison. The data‐acquisition system was used only to record the hardware‐generated binary outputs.

**FIGURE 4:**
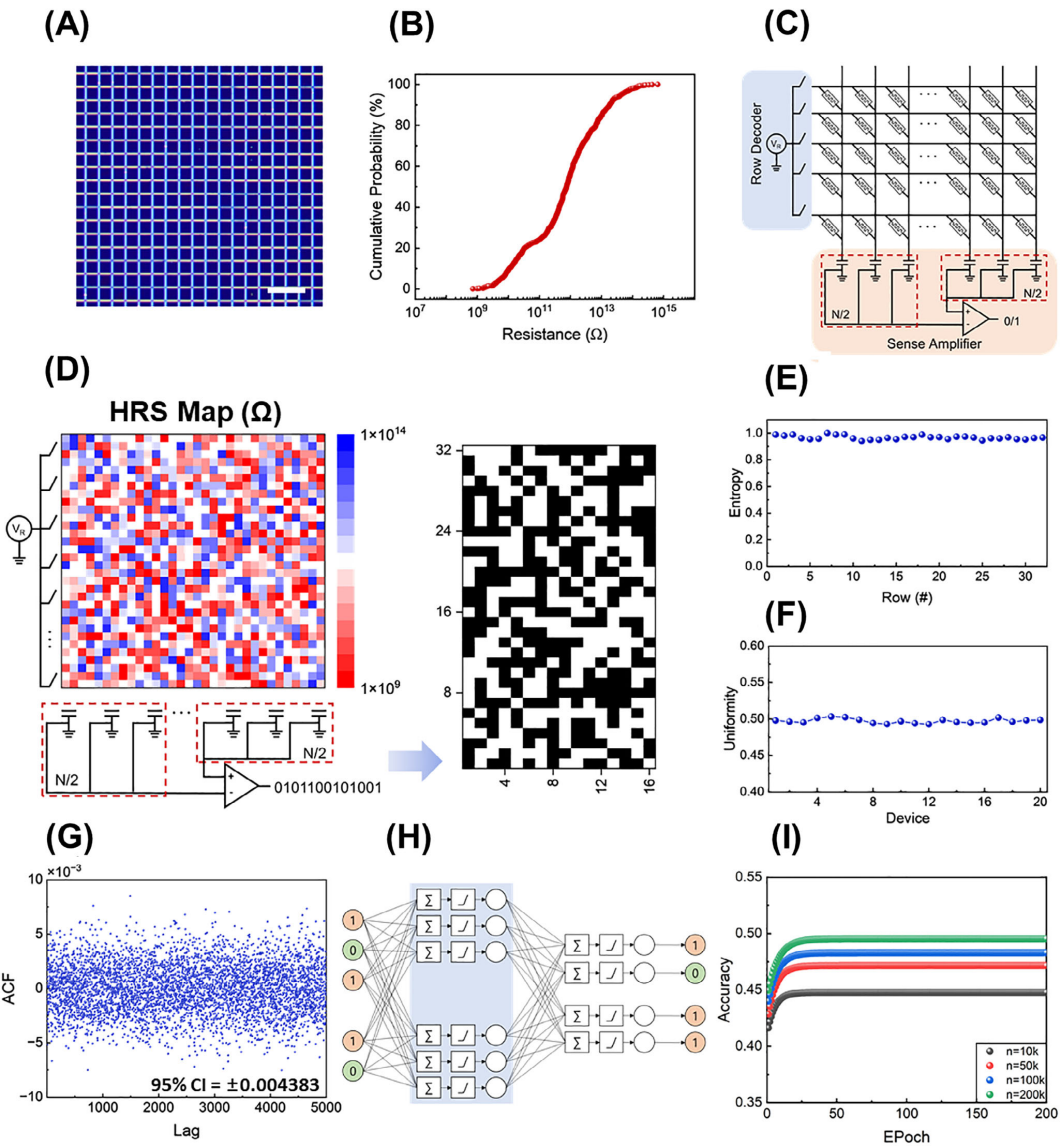
Experimental hardware random‐bit generation and statistical validation in the 32 × 32 memristor array. (A) Microscopic image of the 32 × 32 memristor array (scale bar: 200 µm). (B) Cumulative probability distribution of the HRS current measured at 0.2 V across the array. (C) Schematic circuit architecture illustrating a hardware implementation of paired‐column comparison. (D) Experimentally measured HRS‐current map and corresponding 16‐bit binary map directly acquired from the hardware sense‐amplifier output. (E) Row‐wise entropy of the hardware‐generated bitstream. (F) Bit uniformity measured across 20 independently evaluated devices. (G) Autocorrelation coefficients of the hardware‐generated bitstream. (H) Architecture of the MLP network used for machine‐learning‐based predictability testing. (I) Prediction accuracy of the MLP for the generated bitstream.

A two‐column comparison method was used for hardware random‐bit extraction. The 32 columns in the array were divided into N/2 left and right column groups, and columns with the same index in each group were paired. For each addressed row, the two column currents in each pair were integrated at the corresponding sense nodes and physically compared by the implemented sense amplifier. The resulting binary output followed the same paired‐current decision rule:

(8) $$I_{L,k}=V_{read}\sum_{i=1}^{N}Row_{i}G_{i,k}$$

(9) $$I_{R,k}=V_{read}\sum_{i=1}^{N}Row_{i}G_{i,k+N/2}$$

(10) $$b_{i,k}=\left\{\begin{array}{c} \;0,V_{L,i,k}>V_{R,i,k}\\ 1,V_{L,i,k}<V_{R,ik} \end{array}\right.$$

Here, Row_i_ represents the selection state of the i‐th row and G_i,j_ denotes the device conductance. The implemented readout generates 16 hardware bits per addressed row, and sequential scanning of all 32 rows produces the corresponding binary frame. The long bitstream was accumulated by repeated SET–RESET–read cycles at each fixed row/pair position, with a newly generated post‐RESET HRS state preceding each recorded bit; further experimental details are provided in Figure S9. Figure 4D shows the experimentally measured HRS map and the corresponding hardware‐generated binary map directly acquired from the sense‐amplifier output. The read voltage V_R_ was selected within the linear HRS read regime to minimize read disturbance. Reproducibility was confirmed by repeated hardware scans under identical conditions (Figure S9C,D).

The randomness of the extracted bitstream was evaluated using Shannon entropy [61, 62]. The number of 1s in each 16‐bit word was defined as p, and the entropy E was calculated using:

(11) $$E=-p\log_{2}p\;-\left(1-p\right)\log_{2}\left(1-p\right)$$

The row‐wise entropy of the hardware‐generated output shows an average of 0.967 (Figure 4E). Bit uniformity measured across 20 independently evaluated devices remained close to the ideal value of 0.5, with a mean value of 0.4973 ± 0.0031 and a range of 0.4928–0.5033 (Figure 4F). A NIST SP 800–22 test was performed on the 12.0‐Mbit stream, and all tests were successfully passed; detailed p‐values are provided in Table S1. To evaluate the effect of NI geometry, random bits generated from devices with a 1‐nm NI layer were also analyzed (Figure S10). These bitstreams exhibited an average entropy of 0.941 and a minimum of 0.81. Although comparable to the optimized 1.5‐nm condition, the 1‐nm devices showed slightly greater column‐to‐column variation and a residual bias. This behavior is attributed to the modified nanogap distribution associated with different NI morphologies, further supporting that NI geometry functions as a tunable entropy engine whose physical randomness can be optimized for specific applications such as TRNGs or neuromorphic computing.

To further assess serial dependence after physical bit extraction, the autocorrelation function (ACF) of the hardware‐generated bitstream was evaluated (Figure 4G). The mean autocorrelation coefficient was only 2.3 × 10^−5^, and the maximum absolute coefficient was 0.00852. Only 4.70% of the coefficients fell outside the 95% confidence interval, closely matching the nominal ∼5% crossing rate expected for an uncorrelated sequence.

The bitstreams were also tested against a machine‐learning (ML) attack designed to predict the next bit, b_t+1,_ from the preceding bits. As illustrated in Figure 4H, a multilayer perceptron (MLP) with 32 input neurons, 300 hidden neurons, and 16 output neurons was employed. As shown in Figure 4I, the prediction accuracy saturated below 0.5 even with increasing training samples, indicating that the MLP was unable to learn any meaningful statistical patterns. These results confirm that the proposed method achieves both high entropy and low correlation in a 32 × 32 array. Compared with representative memristor‐based TRNGs (Table S2), the present platform combines a morphology‐defined physical entropy source, paired‐column differential extraction, 32 × 32 array‐level implementation, 10^5^‐cycle endurance at both 25°C and 85°C, and statistical validation using a 12.0‐Mbit stream. Conventional filamentary ReRAMs can likewise exploit reset‐dependent HRS variability for random‐bit generation, and statistically validated TRNG operation can be achieved through appropriate electrical programming and extraction schemes. The present Au‐NI strategy is therefore not intended to replace such electrical optimization or to imply universally superior TRNG performance. Instead, it introduces a complementary materials‐level design axis in which the stochastic HRS landscape can be modulated through the device morphology itself. Under the same reset/read protocol, the NI‐thickness series exhibits systematic changes in HRS dispersion and recovery, with NI‐1.5 providing a broad reproducible HRS distribution while retaining reliable recovery, whereas excessive coalescence at NI‐2.0 results in degraded HRS recovery. This behavior indicates that morphology can be used to preset the accessible entropy–operability regime during fabrication, in addition to subsequent electrical control. Such a materials‐level design degree of freedom may reduce reliance on aggressive or device‐specific reset‐condition optimization, although electrical tuning is not eliminated. The approach also introduces an additional ultrathin Au‐deposition and morphology‐control requirement, and excessive coalescence represents an experimentally observed trade‐off. Thus, the practical significance of the Au‐NI approach lies in extending entropy engineering from operating‐condition optimization alone toward joint materials‐ and operation‐level control of stochasticity, recovery, reproducibility, and array operability. A focused comparison with representative HRS‐variability‐based filamentary ReRAM TRNGs is provided in Table S4.

To quantitatively summarize the morphology‐dependent entropy‐engineering trend, the extracted morphology descriptors and array‐level randomness metrics are integrated in Figure 5A–C. Figure 5A presents comparative morphology descriptors obtained from the AFM‐derived relative β analysis. The β‐distribution width and hotspot density, defined here as the high‐β area fraction, quantify the relative spatial variation and areal population of pronounced values within the normalized field‐concentration proxy. Both descriptors increase from NI‐0.5 to NI‐1.5, indicating progressive broadening of the morphology‐derived field‐concentration landscape. These quantities are comparative descriptors and are not interpreted as measured absolute electric‐field enhancement factors or quantitative filament‐nucleation probabilities.

**FIGURE 5:**
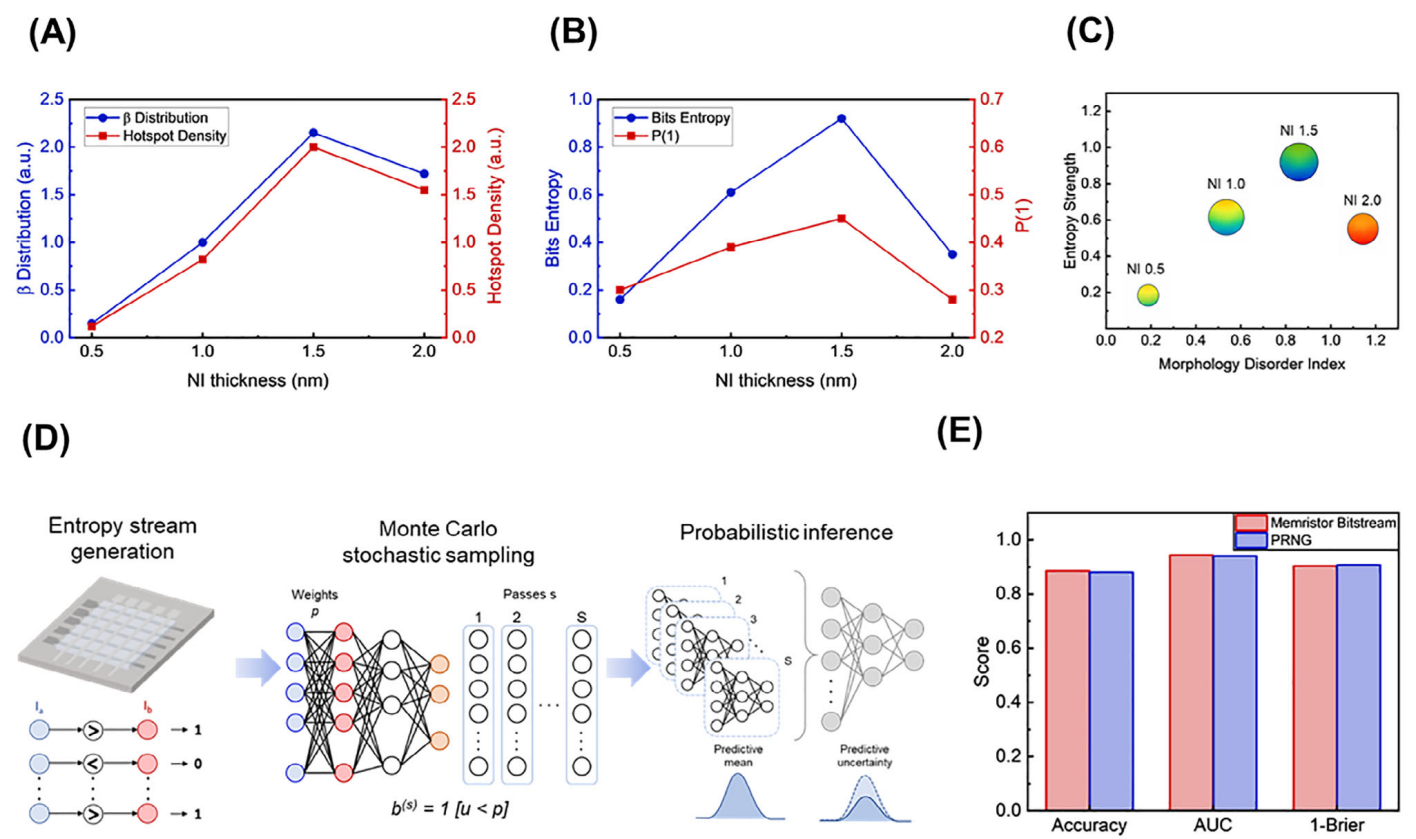
Morphology‐guided entropy engineering and Monte Carlo sampling validation in memristor arrays. (A) Morphology‐derived hotspot descriptors, including β‐distribution width and hotspot density, obtained from the field‐amplification analysis. Both descriptors increase from NI‐0.5 to NI‐1.5, indicating the progressive broadening of the morphology‐derived field‐concentration‐proxy landscape. (B) Array‐level randomness metrics of the generated bitstreams. Bit entropy, min‐entropy, and output uniformity improve up to NI‐1.5, showing a systematic association between the morphology‐derived descriptors, HRS‐current dispersion, and array‐level randomness quality. (C) Integrated morphology–entropy map based on the normalized morphology disorder index and entropy strength index. The morphology disorder index combines β‐distribution width and hotspot density, while the entropy strength index combines bit entropy, min‐entropy, and output uniformity. NI‐1.5 is identified as a balanced entropy‐engineering window, whereas NI‐2.0 corresponds to an over‐coalesced, breakdown‐prone regime. These results identify NI‐1.5 as a balanced entropy‐engineering regime in which broad morphology‐derived descriptors, enhanced HRS stochasticity, and reliable HRS recovery are simultaneously observed. (D) Workflow for extending the statistically validated memristor‐derived entropy stream to Monte Carlo probabilistic inference. (E) Sampling‐fidelity validation of the memristor‐derived entropy stream in Monte Carlo probabilistic inference.

Figure 5B summarizes the array‐level randomness metrics extracted from the generated bitstreams. The entropy strength index was defined as a normalized descriptor combining bit entropy, min‐entropy, and output uniformity, thereby capturing the average randomness, worst‐case predictability, and bias balance of the generated bitstream. The increase in entropy strength from NI‐0.5 to NI‐1.5 occurs together with the progressive broadening of the morphology‐derived field‐concentration descriptors and the experimentally observed HRS‐current dispersion, revealing a systematic correlation among Au‐NI morphology, HRS switching statistics, and array‐level randomness quality. In contrast, NI‐2.0 does not further improve the usable entropy window despite its increased structural continuity, because over‐coalescence promotes poor HRS recovery and breakdown‐prone conduction.

By combining these descriptors, Figure 5C constructs an integrated morphology–entropy map based on the normalized morphology disorder index and entropy strength index. The morphology disorder index was calculated from the β‐distribution width and hotspot density, whereas the entropy strength index was calculated from bit entropy, min‐entropy, and output uniformity. The map identifies NI‐1.5 as a balanced entropy‐engineering window, where hotspot diversity, HRS recoverability, and bit‐generation quality are simultaneously satisfied. The morphology–entropy map therefore identifies NI‐1.5 as a balanced entropy‐engineering regime in which broad morphology‐derived descriptors coincide with enhanced HRS stochasticity, reliable HRS recovery, and high bit‐generation quality.

Having identified NI‐1.5 as the balanced morphology–entropy design window, we next extend the role of the validated entropy stream from statistical random‐bit generation to stochastic sampling, evaluating whether morphology‐defined HRS stochasticity can support probability‐aware Monte Carlo inference. Figure 5D illustrates the workflow for using the validated memristor‐derived entropy stream as a stochastic sampling primitive for Monte Carlo probabilistic inference; the detailed protocol is described in Note S1. The HRS‐current dispersion in the memristor array is converted into an entropy stream with high entropy, balanced bit probability, and low autocorrelation through paired‐current comparison. This stream then drives stochastic binary weight‐sampling events, generating a different weight configuration for each Monte Carlo pass. The outputs from repeated stochastic forward passes are aggregated as an ensemble, from which the predictive mean and predictive uncertainty are obtained. This use of device‐derived stochasticity as a sampling resource is consistent with recent probabilistic neural‐computing frameworks based on stochastic devices and emerging memristive hardware [63, 64, 65]. In this way, the memristor‐derived stream extends beyond random‐bit output and serves as a physical entropy source that supports the sampling operations required for probabilistic computing.

Figure 5E quantitatively summarizes the resulting entropy‐stream‐driven inference. When the memristor bitstream was used as the sampling source, the inference achieved an accuracy of 0.8856, an AUC of 0.9431, and a 1−Brier score of 0.9037. These results indicate that stable decision accuracy and high class separability are retained even under stochastic sampling, while the probabilistic outputs remain well aligned with the ground‐truth labels. In particular, because the 1−Brier score is directly related to the calibration of output probabilities, the result supports that the memristor‐derived entropy stream can provide the sampling fidelity required for probability‐aware inference, beyond simple bit generation. Taken together, the results demonstrate that morphology‐defined HRS stochasticity can be extended from array‐level entropy validation to Monte Carlo probabilistic computing.

Table S3 compares the present platform with recent stochastic memristive computing devices in which device‐level stochasticity is used for information processing. Whereas previous studies have primarily focused on mapping device stochasticity to specific probabilistic computing primitives, such as stochastic bits, probabilistic weights, p‐bits, or stochastic number encoders, this work controls the physical distribution and entropy quality of the stochastic source itself through Au NI morphology and links it to array‐level validation and Monte Carlo sampling fidelity. Thus, the distinction of this work lies not in the use of stochasticity alone, but in transforming morphology‐defined HRS‐current dispersion into a designable entropy resource that can support both statistical randomness and probabilistic sampling. These results show that structural disorder does not necessarily remain a source of memristor variability; when properly engineered, it can serve as a materials‐level control parameter that determines the quality of a physical entropy stream. The Au NI morphology‐engineered memristor array therefore provides a stochastic information platform that bridges hardware randomness and Monte Carlo probabilistic sampling, offering a design guideline for integrating morphology‐defined entropy sources into uncertainty‐aware computing and probabilistic hardware.

## 3 Conclusion

This work demonstrates that Au NI morphology can modulate the statistical characteristics of stochastic HRS transport in electrochemical memristors and thereby engineer the quality of a physical entropy stream. The observed stochastic transport does not originate exclusively from Au NI morphology, but from the coupled action of morphology‐related field heterogeneity, residual filament configurations, local defects, trap states, and stochastic ionic motion. Within this multivariable process, the Au NI morphology functions as a designable structural control parameter that tunes the distribution and recoverability of HRS states. The systematic evolution from weak stochasticity in NI‐0.5, through the balanced NI‐1.5 regime, to breakdown‐prone behavior in NI‐2.0 supports this morphology‐dependent modulation. The entropy stream extracted from the 32 × 32 array was directly converted into binary outputs using an experimentally implemented readout and statistically validated using bit probability, binary entropy, autocorrelation, NIST SP 800–22 tests, and machine‐learning‐based predictability analysis. The hardware‐generated stream retained high entropy, near‐ideal bit balance, and negligible serial correlation, establishing the experimental link between device‐level stochastic HRS transport and physical random‐bit generation. These results show that the HRS fluctuations systematically associated with Au NI morphology are not merely undesirable variability, but can function as an array‐level physical entropy source. Au NI morphology is therefore treated as a designable structural control parameter that modulates the statistical characteristics and recoverability of HRS states within a multivariable stochastic switching process. By further applying the validated entropy stream to Monte Carlo stochastic sampling, we show that the stream can be extended beyond random‐bit generation as a sampling primitive for probabilistic information processing. The central claim is the demonstration that morphology‐defined stochastic transport can serve as a physical information resource supporting both statistical randomness and sampling fidelity. This study therefore reframes nanoscale structural disorder not solely as a source of reliability degradation, but as a controllable entropy resource. By engineering HRS stochasticity through Au NI morphology and connecting it to array‐level entropy validation and Monte Carlo probabilistic sampling, this work suggests a pathway for extending stochastic memristor arrays into materials‐defined entropy platforms for hardware randomness, uncertainty‐aware sampling, and probabilistic information processing.

## 4 Experimental Section/Methods

### 4.1 Device Fabrication

The devices were fabricated on a heavily doped p^++^ silicon substrate using standard semiconductor processing; the fabrication flow is shown in Figure S1. Substrates were cleaned sequentially with acetone, isopropyl alcohol (IPA), and deionized (DI) water to remove organic contaminants. The bottom electrode was deposited by e‐beam evaporation (5 nm Ti adhesion layer and 50 nm Pt) and patterned by a lift‐off process. Au NI interlayers with nominal mass‐equivalent thicknesses of 0.5, 1.0, 1.5, and 2.0 nm were deposited onto the Pt bottom electrode by electron‐beam evaporation using a 99.99% Au source. The chamber base pressure was approximately 1 × 10^−6^ Torr, and the deposition rate was maintained at 0.1 Å s^−1^. The deposition rate and accumulated nominal thickness were monitored in real time using a quartz crystal microbalance. The QCM tooling factor was set to 100%, and no additional empirically determined geometric correction was applied between the sensor and substrate positions. The substrate shutter was opened after stabilization of the evaporation rate, and deposition was terminated upon reaching the target nominal mass‐equivalent thickness. The reported Au thicknesses therefore represent QCM‐monitored nominal mass‐equivalent values rather than the local physical heights of the three‐dimensional Au NI. After formation of the Au NI, a 20‐nm‐thick TiO_2_ layer was deposited by electron‐beam evaporation directly from a commercial TiO_2_ ceramic pellet. Prior to deposition, the chamber was evacuated to a base pressure of approximately 1 × 10^−^^6^ Torr, and the deposited thickness was monitored in real time using a quartz crystal microbalance (QCM). No intentional oxygen was introduced during deposition, and no post‐deposition oxidation or thermal annealing was performed. Finally, an Ag top electrode was deposited and patterned using the same lift‐off procedure.

### 4.2 Characterization

Top‐view images of the Au NIs were acquired by field‐emission scanning electron microscopy (FE‐SEM; Hitachi). Cross‐sectional images of the full device stack were obtained by high‐resolution scanning transmission electron microscopy (HR‐STEM). To investigate the structural evolution of Au NI growth during evaporation, glancing‐angle x‐ray diffraction (GIXRD; Bruker AXS) was performed at room temperature using Cu Kα radiation over a 2θ range of 20°–80°. Electrical measurements were carried out with a Keithley 4200 semiconductor parameter analyzer connected to a probe station (MS Tech, Model 4000); voltage bias was applied to the top electrode while the bottom electrode was grounded. The chemical state of the as‐deposited TiO_2_ layer was further examined by x‐ray photoelectron spectroscopy (XPS) using a companion film deposited under the same electron‐beam evaporation conditions as those used for the memristor devices.

## Author Contributions

**Hyunjun Kim**: investigation, methodology, data curation , formal analysis, visualization. **Da Ye Kim**: investigation, data curation, writing – original draft, software. **Noah Jang**: methodology, data curation, writing – original draft, validation. **Yongho Lee**: investigation, data curation. **Byunghun Song**: investigation, methodology. **June Soo Kim**: conceptualization, supervision, writing – review and editing, project administration, resources, funding acquisition.

## Conflicts of Interest

The authors declare no conflicts of interest.

## Supporting information




**Supporting File**: advs78052‐sup‐0001‐SuppMat.docx.

## Data Availability

The data that support the findings of this study are available from the corresponding author upon reasonable request.

## References

[1] X. Zhao , L.‐W. Chen , K. Li , H. Schmidt , I. Polian , and N. Du , “Memristive True Random Number Generator for Security Applications,” Sensors 24, no. 15 (2024): 5001, 10.3390/s24155001. PMC11314823 39124048

[2] J. Kelsey , B. Schneier , D. Wagner , and C. Hall , Fast Software Encryption—FSE’98, 5th International Workshop, Proceedings (Springer, 1998), 168–188, 10.1007/3-540-69710-1_12.

[3] S. J. Oks , M. Jalowski , M. Lechner , et al., “Cyber‐Physical Systems in the Context of Industry 4.0: A Review, Categorization and Outlook,” Information Systems Frontiers 24 (2022): 731–1772, 10.1007/s10796-022-10252-x.

[4] M. S. Farooq , M. Abdullah , S. Riaz , et al., “A Survey on the Role of Industrial IoT in Manufacturing for Implementation of Smart Industry,” Sensors 23, no. 21 (2023): 8958, 10.3390/s23218958. PMC10650216 37960657

[5] I. Goodfellow , J. Pouget‐Abadie , M. Mirza , et al., “Generative Adversarial Nets,” Advances in Neural Information Processing Systems 27 (2014): 2672.

[6] J. Ho , A. Jain , and P. Abbeel , “Denoising Diffusion Probabilistic Models,” Advances in Neural Information Processing Systems 33 (2020): 6840, 10.48550/arXiv.2006.11239.

[7] R. Rombach , A. Blattmann , D. Lorenz , et al., “High‐Resolution Image Synthesis With Latent Diffusion Models,” in 2022 IEEE/CVF Conference on Computer Vision and Pattern Recognition (IEEE, 2022): 10684–10695, 10.1109/CVPR52688.2022.01042.

[8] I. Rivero‐García , M. Torres , and F. Sánchez‐Cabo , “Deep Generative Models in Single‐cell Omics,” Computer in Biology Medicine 176 (2024): 108561, 10.1016/j.compbiomed.2024.108561. 38749321

[9] M. Nishio , C. Muramatsu , S. Noguchi , et al., “Attribute‐Guided Image Generation of Three‐Dimensional Computed Tomography Images of Lung Nodules Using a Generative Adversarial Network,” Computers in Biology and Medicine 126 (2020): 104032, 10.1016/j.compbiomed.2020.104032. 33045649

[10] N. Bochard , V. Fischer , F. Bernard , and B. Valtchanov , “True‐Randomness and Pseudo‐Randomness in Ring Oscillator‐Based True Random Number Generators,” International Journal of Reconfigurable Computing 2010 (2010): 1–13, 10.1155/2010/879281.

[11] C. S. Petrie and J. A. Connelly , “A Noise‐Based IC Random Number Generator for Applications in Cryptography,” IEEE Transactions on Circuits and Systems I: Fundamental Theory and Applications 47, no. 5 (2000): 615–621, 10.1109/81.847868.

[12] H. J. Ng , S. Yang , Z. Yao , H. Yang , and C. Lim , “Provably Secure Randomness Generation From Switching Probability of Magnetic Tunnel Junctions,” Physical Review Applied 19, no. 3 (2023): 034077, 10.1103/PhysRevApplied.19.034077.

[13] S. Jape , S. Patil , S. Patil , and N. Kulkarni , “Design of a Novel Non‐Volatile Hybrid Spintronic True Random Number Generator,” International Journal of Circuit Theory and Applications 50, no. 5 (2022): 1487–1501, 10.1002/cta.3243.

[14] K. S. Woo , A. Zhang , A. Arabelo , et al., “True Random Number Generation Using the Spin Crossover in LaCoO_3_ ,” Nature Communications 15, no. 1 (2024): 4656, 10.1038/s41467-024-44656-y. PMC11143320 38821970

[15] H. Jiang , D. Belkin , S. E. Savel'ev , et al., “A Novel True Random Number Generator Based on a Stochastic Diffusive Memristor,” Nature Communications 8, no. 1 (2017): 882, 10.1038/s41467-017-00869-x. PMC5638922 29026110

[16] S. Pazos , W. Zheng , T. Zanotti , et al., “Hardware Implementation of a True Random Number Generator Integrating a Hexagonal Boron Nitride Memristor With a Commercial Microcontroller,” Nanoscale 15, no. 5 (2023): 2171–2180, 10.1039/D2NR06222D. 36628646

[17] Y. Pang , B. Gao , B. Lin , H. Qian , and H. Wu , “Memristors for Hardware Security Applications,” Advanced Electronic Materials 5, no. 9 (2019): 1800872, 10.1002/aelm.201800872.

[18] Y. Wang , N. Xu , Y. Yuan , et al., “Achieving Adjustable Digital‐to‐analog Conversion in Memristors with Embedded Cs^2^agsbbr₆ Nanoparticles,” Nanoscale 15 (2023): 7344–7351, 10.1039/D2NR06370K. 37038924

[19] S. I. Kim , H.‐J. You , M.‐S. Kim , et al., “Cryptographic Transistor for True Random Number Generator With Low Power Consumption,” Science Advances 10 (2024): adk6042, 10.1126/sciadv.adk6042. PMC10889348 38394200

[20] U. Welzel , J. Ligot , P. Lamparter , A. C. Vermeulen , and E. J. Mittemeijer , “Stress Analysis of Polycrystalline Thin Films and Surface Regions by X‐Ray Diffraction,” Journal of Applied Crystallography 38, no. 1 (2005): 1–29, 10.1107/S0021889804029516.

[21] G. F. Harrington and J. Santiso , “Back‐to‐Basics Tutorial: X‐Ray Diffraction of Thin Films,” Journal of Electroceramics 47, no. 4 (2021): 141–163, 10.1007/s10832-021-00263-6.

[22] F. T. L. Muniz , M. A. R. Miranda , C. M. dos Santos , and J. M. Sasaki , “The Scherrer Equation and the Dynamical Theory of X‐Ray Diffraction,” Acta Crystallographica Section A Foundations and Advances 72, no. 3 (2016): 385–390, 10.1107/S205327331600365X. 27126115

[23] D. I. Yakubovsky , A. V. Arsenin , Y. V. Stebunov , D. Y. Fedyanin , and V. S. Volkov , “Optical Constants and Structural Properties of Thin Gold Films,” Optics Express 25, no. 21 (2017): 25574, 10.1364/OE.25.025574. 29041223

[24] E. Irissou , M.‐C. Denis M. Chaker , and D. Guay , “Gold Oxide Thin Film Grown by Pulsed Laser Deposition in an O^2^ Atmosphere,” Thin Solid Films 472 (2005): 49–57, 10.1016/j.tsf.2004.06.092.

[25] J. Hu , W. Li , J. Chen , X. Zhang , and X. Zhao , “Novel Plating Solution For Electroless Deposition of Gold Film Onto Glass Surface,” Surface & Coating Technology 202 (2008): 2922–2926, 10.1016/j.surfcoat.2007.10.026.

[26] G. M. Feeley , J. M. White , and C. T. Campbell , “Energetics of Au Adsorption and Film Growth on Pt(111) by Single‐Crystal Adsorption Calorimetry,” The Journal of Physical Chemistry C 123, no. 9 (2019): 5557–5561, 10.1021/acs.jpcc.9b00018.

[27] K. Krupski , M. Moors , P. Jozwik , T. Kobiela , and A. Krupski , “Structure Determination of Au on Pt(111) Surface: LEED, STM and DFT Study,” Materials 8, no. 6 (2015): 2935–2952, 10.3390/ma8062935.

[28] A. Trembułowicz , A. Sabik , L. Jurczyszyn , and S. Rep , “Structural and Electronic Properties of Pt Modified Au (100) Surface,” Scientific Reports 12 (2022): 3859, 10.1038/s41598-022-07617-2. PMC8907180 35264635

[29] Y. M. Koroteev , I. V. Silkin , V. M. Silkin , and E. V. Chulkov , “Quantum‐Size Effects in Ultra‐Thin Gold Films on Pt(111) Surface,” Materials 17 (2024): 63, 10.3390/ma17010063. PMC10779727 38203917

[30] J. A. Venables , “Nucleation and Growth of Thin Films,” Reports on Progress in Physics 47, no. 4 (1984): 399–459, 10.1088/0034-4885/47/4/002.

[31] H. Brune , “Microscopic View of Epitaxial Metal Growth: Nucleation and Aggregation,” Surface Science Reports 31, no. 4‐6 (1998): 125–229, 10.1016/S0167-5729(99)80001-6.

[32] M. H. Grabow and G. H. Gilmer , “Thin Film Growth Modes, Wetting and Cluster Nucleation,” Surface Science 194, no. 3 (1988): 333–346, 10.1016/0039-6028(88)90858-8.

[33] Z. Zhang and M. G. Lagally , “Atomistic Processes in the Early Stages of Thin‐Film Growth,” Science 276, no. 5311 (1997): 377–383, 10.1126/science.276.5311.377. 9103189

[34] M. Nishio , C. Muramatsu , S. Noguchi , et al., “Attribute‐guided Image Generation of Three‐dimensional Computed Tomography Images of Lung Nodules Using a Generative Adversarial Network,” Computers in Biology Medicine 126 (2020): 104032, 10.1016/j.compbiomed.2020.104032. 33045649

[35] C. Ratsch and J. A. Venables , “Nucleation Theory and the Early Stages of Thin Film Growth,” Journal of Vacuum Science & Technology, A: Vacuum, Surfaces, and Films 21 (2003): S96–S109, 10.1116/1.1600454.

[36] A. Pimpinelli , L. Tumbek , and A. Winkler , “Scaling and Exponent Equalities in Island Nucleation: Novel Results and Application to Organic Films,” The Journal of Physical Chemistry Letters 5, no. 6 (2014): 995–998, 10.1021/jz500282t. PMC3962253 24660052

[37] P. A. Thiel and M. Shen , “Nucleation, Growth, and Relaxation of Thin Films: Metal(100) Homoepitaxial Systems,” The Journal of Physical Chemistry B 104, no. 8 (2000): 1663–1676, 10.1021/jp9933471.

[38] I. Valov , R. Waser , J. R. Jameson , and M. N. Kozicki , “Electrochemical Metallization Memories—Fundamentals, Applications, Prospects,” Nanotechnology 22, no. 25 (2011): 254003, 10.1088/0957-4484/22/25/254003. 21572191

[39] D. Ielmini , “Resistive Switching Memories Based on Metal Oxides: Mechanisms, Reliability and Scaling,” Semiconductor Science and Technology 31, no. 6 (2016): 063002, 10.1088/0268-1242/31/6/063002.

[40] D. A. Shiffler , W. Tang , K. L. Jensen , et al., “Effective Field Enhancement Factor and the Influence of Emitted Space Charge,” Journal of Applied Physics 118, no. 8 (2015): 083302, 10.1063/1.4929461.

[41] R. G. Forbes and J. H. B. Deane , “Physical Electrostatics of Small Field Emitter Arrays/Clusters,” Journal of Applied Physics 108 (2010): 044502, 10.1063/1.3467520.

[42] G. Bocharov and A. Eletskii , “Theory of Carbon Nanotube (CNT)‐Based Electron Field Emitters,” Nanomaterials 3, no. 3 (2013): 393–442, 10.3390/nano3030393. PMC5304654 28348342

[43] S. Menzel , S. Tappertzhofen , R. Waser , and I. Valov , “Switching Kinetics of Electrochemical Metallization Memory Cells,” Physical Chemistry Chemical Physics 15, no. 18 (2013): 6945, 10.1039/C3CP50738F. 23549450

[44] S. A. Chekol , S. Menzel , R. Waser , and S. Hoffmann‐Eifert , “Strategies to Control the Relaxation Kinetics of Ag‐Based Diffusive Memristors and Implications for Device Operation,” Advanced Electronic Materials 8, no. 11 (2022): 2111242, 10.1002/aelm.202111242.

[45] M. Buttberg , I. Valov , and S. Menzel , “Simulating the Filament Morphology in Electrochemical Metallization Cells,” Neuromorphic Computing and Engineering 3, no. 2 (2023): 024010, 10.1088/2634-4386/acc6a0.

[46] B. K. You , J. M. Kim , D. J. Joe , et al., “Reliable Memristive Switching Memory Devices Enabled by Densely Packed Silver Nanocone Arrays as Electric‐Field Concentrators,” ACS Nano 10, no. 10 (2016): 9478–9488, 10.1021/acsnano.6b04578. 27718554

[47] S. Dirkmann , M. Ziegler , M. Hansen , H. Kohlstedt , J. Trieschmann , and T. Mussenbrock , “Kinetic Simulation of Filament Growth Dynamics in Memristive Electrochemical Metallization Devices,” Journal of Applied Physics 118, no. 21 (2015): 214501, 10.1063/1.4936599.

[48] O. Kapur , D. Guo , J. Reynolds , et al., “An Ultra High‐Endurance Memristor Using Back‐End‐Of‐Line Amorphous SiC,” Scientific Reports 14, no. 1 (2024): 14008, 10.1038/s41598-024-64499-2. PMC11189404 38890324

[49] N. Carstens , A. Vahl , A. Vahl , et al., “Enhancing Reliability of Studies on Single Filament Memristive Switching via an Unconventional cAFM Approach,” Nanomaterials 11 (2021): 265, 10.3390/nano11020265. PMC7909531 33498494

[50] L. Li , W. Fa , and S. Chen , “Computational Study on Filament Growth Dynamics in Microstructure‐Controlled Storage Media of Resistive Switching Memories,” ACS Nano 17 (2023): 10266, 10.1021/acsnano.3c01405. 37235757

[51] K. Min , D. Jung , and Y. Kwon , “Investigation of Switching Uniformity in Resistive Memory via Finite Element Simulation of Conductive‐Filament Formation,” Scientific Reports 11 (2021): 2447, 10.1038/s41598-021-81896-z. PMC7843989 33510234

[52] K. Zhang , P. Ganesh , and Y. Cao , “Deterministic Conductive Filament Formation and Evolution for Improved Switching Uniformity in Embedded Metal‐Oxide‐Based Memristors─A Phase‐Field Study,” ACS Applied Materials & Interfaces 15, no. 17 (2023): 21219–21227, 10.1021/acsami.3c00371. 37083295

[53] J. Xu , L. Qiao , Y. Yang , et al., “NbOx RRAM Performance Enhancement by Surface Modification With Au Nanoparticles,” Vacuum 227 (2024): 113422, 10.1016/j.vacuum.2024.113422.

[54] R. H. Fowler and L. Nordheim , “Electron Emission in Intense Electric Fields,” Proceedings of the Royal Society of London Series A, Containing Papers of a Mathematical and Physical Character 119, no. 781 (1928): 173–181, 10.1098/rspa.1928.0091.

[55] J. G. Simmons , “Generalized Formula for the Electric Tunnel Effect Between Similar Electrodes Separated by a Thin Insulating Film,” Journal of Applied Physics 34, no. 6 (1963): 1793–1803, 10.1063/1.1702682.

[56] E. W. Lim and R. Ismail , “Conduction Mechanism of Valence Change Resistive Switching Memory: A Survey,” Electronics 4, no. 3 (2015): 586–613, 10.3390/electronics4030586.

[57] C. Funck and S. Menzel , “Comprehensive Model of Electron Conduction in Oxide‐Based Memristive Devices,” ACS Applied Electronic Materials 3 (2021): 3674–3692, 10.1021/acsaelm.1c00398.

[58] R. G. Forbes and J. H. B. Deane , “Reformulation of the Standard Theory of Fowler–Nordheim Tunnelling and Cold Field Electron Emission,” Proceedings of Royal Society of London Series A 463 (2007): 2907, 10.1098/rspa.2007.0030.

[59] S. Samanta , S. Z. Rahaman , A. Roy , et al., “Understanding of Multi‐Level Resistive Switching Mechanism in GeOx Through Redox Reaction in H_2_O_2_/Sarcosine Prostate Cancer Biomarker Detection,” Scientific Reports 7, no. 1 (2017): 11739, 10.1038/s41598-017-11657-4. PMC5593955 28894240

[60] Z. Ma , J. Ge , W. Chen , et al., “Analog Tunnel Memory Based on Programmable Metallization for Passive Neuromorphic Circuits,” ACS Applied Materials & Interfaces 14, no. 42 (2022): 47941–47951, 10.1021/acsami.2c14809. 36223072

[61] C. E. Shannon , “A Mathematical Theory of Communication,” Bell System Technical Journal 27, no. 3 (1948): 379–423, 10.1002/j.1538-7305.1948.tb01338.x.

[62] T. M. Cover and J. A. Thomas , Elements of Information Theory (Wiley, 2006), 10.1002/047174882X.

[63] S. Misra , L. C. Bland , S. G. Cardwell , et al., “Probabilistic Neural Computing With Stochastic Devices,” Advanced Materials 35, no. 37 (2023): 2204569, 10.1002/adma.202204569. 36395387

[64] Y. Zheng , A. Ravichandran , N. Trainor , J. M. Redwing , and S. Das , “Hardware Implementation of Bayesian Network Based on Two‐Dimensional Memtransistors,” Nature Communications 13, no. 1 (2022): 5578, 10.1038/s41467-022-33053-x. PMC9508127 36151079

[65] D. Bonnet , G. Bourgeois , J. M. Portal , D. Querlioz , and E. Vianello , “Bringing Uncertainty Quantification to the Extreme‐Edge With Memristor‐Based Bayesian Neural Networks,” Nature Communications 14, no. 1 (2023): 7530, 10.1038/s41467-023-43317-9. PMC10661910 37985669

