# Supplementary material for: Morphology‐Defined Entropy Streams for Random‐Bit Generation and Probabilistic Sampling in Memristor Arrays
Source: Adv Sci (Weinh). 2026 Oct 8:e78052. Online ahead of print. doi: 10.1002/advs.78052 (PMC13650020; doi:10.1002/advs.78052)
Supplement: Supplementary file 1 [file ADVS-9999-e78052-s001.docx]

Supplementary Materials for

Morphology-Defined Entropy Streams for Random-Bit Generation and Probabilistic Sampling in Memristor Arrays

*Hyunjun Kim*‡*, Da Ye Kim*‡*, Noah Jang*‡*, Yongho Lee, Byunghun Song, and June Soo Kim**

Hyunjun Kim, Da Ye Kim, Noah Jang

School of Electronic and Electrical Engineering, Kyungpook National University, Daegu, 41566, Republic of Korea

Yongho Lee, Byunghun Song

Smart Manufacturing Innovation Centre, Korea Electronics Technology Institute, Seongnam, 13509, Republic of Korea

June Soo Kim

Department of Electronic Engineering, Cheongju University, 298 Daesung-ro, Cheongju 28503, Republic of Korea

**Supplementary Figures and Tables**

**Figure S1.** **(A)** Fabrication process of the Ag/TiO_2_/Au NI/Pt memristor array. **(B)** EDS elemental mapping of the memristor device.

**Figure S2. (A)** Top-view SEM images of Au NI deposited with thicknesses from 0.5 to 2 nm with 4 respective deposition process for each condition (scale bar: 50 nm). **(B)** Calculated NI diameters and **(C)** calculated NI heights for each deposition thickness. **(D)** High-resolution Ti 2p spectrum showing Ti 2p₃/₂ and Ti 2p₁/₂ peaks at approximately 458.5 and 464.2 eV, respectively, with a spin–orbit separation of approximately 5.7 eV. **(E)** High-resolution O 1s spectrum showing the principal oxygen peak at approximately 529.8 eV.

**Figure S3.** TCR analysis of the memristor compared with a pristine TiO_2_-based ECM memristor.

**Figure S4.** Delay time versus 1/V for the memristor measured at different temperatures.

**Figure S5.** Endurance and retention characteristics of the memristor at **(A)** 25 °C and **(B)** 85 °C.

**Figure S6.** NI-thickness-dependent switching variability and device-to-device statistics.

**Figure S7.** **(A)** Calculation procedure for electric-field amplification based on the AFM image. **(B)** AFM image of the NI distribution (1 µm × 1 µm field of view). Heatmaps of **(C)** β and **(D)** κ showing strong electric-field concentration at two characteristic high-curvature regions: the NI summit and rim.

**Figure S8.** Trend of HRS variation as a function of reset amplitude.

**Figure S9.** **(A)** Schematic illustration and **(B)** photograph of the experimentally implemented capacitor-integration/sense-amplifier readout circuit for paired-column comparison. **(C)** Twenty representative hardware-generated binary maps obtained from repeated readout operations of the memristor array. **(D)** Row-wise entropy statistics obtained from the repeated hardware acquisitions.

**Figure S10. (A)** Resistance map and extracted bits from the memristor array with NI = 1.0 nm. **(B)** Entropy distribution for each row.

**Figure S11**. Voltage-based FN representations of memristor with different temperature.

**Supplementary Note 1.** Monte Carlo stochastic sampling protocol using the memristor-derived bitstream.

**Supplementary Note 2.** Interpretation of the FN-Derived Effective Transport-Gap Parameter

**Table S1.** NIST SP 800-22 test results for the generated random bits.

**Table S2. Benchmark comparison of representative memristor-based true random number generators using key device- and randomness-related metrics.**

**Table S3.** Comparison with recent stochastic memristive computing devices.

**Table S4.** Comparison with representative HRS-variability-based filamentary ReRAM TRNGs

**Figure S1.** **(A)** Fabrication process of the Ag/TiO_2_/Au NI/Pt memristor array. **(B)** EDS elemental mapping of the memristor device.

**Figure S2.** **(A)** Top-view SEM images of Au NI deposited with thicknesses from 0.5 to 2 nm with 4 respective deposition process for each condition (scale bar: 50 nm). **(B)** Calculated NI diameters and **(C)** calculated NI heights for each deposition thickness. **(D)** High-resolution Ti 2p spectrum showing Ti 2p₃/₂ and Ti 2p₁/₂ peaks at approximately 458.5 and 464.2 eV, respectively, with a spin–orbit separation of approximately 5.7 eV. **(E)** High-resolution O 1s spectrum showing the principal oxygen peak at approximately 529.8 eV. All binding energies were charge-referenced to the adventitious C 1s peak at 284.8 eV. The measured peak positions are consistent with a Ti^4+^–O-dominated titanium oxide chemical state. These results indicate that the as-deposited titanium oxide layer is dominated by Ti^4+^–O bonding. No pronounced lower-binding-energy shoulder associated with a substantial Ti^3+^ contribution was evident in the measured Ti 2p spectrum. RSF-corrected quantification yielded 61.24 at.% O, 27.44 at.% Ti, and 11.32 at.% adventitious C, corresponding to an O/Ti atomic ratio of 2.23.

**Figure S3.** TCR analysis of the memristor compared with a pristine TiO_2_-based ECM memristor.

**Figure S4.** Delay time versus 1/V for the memristor measured at different temperatures.

**Figure S5.** Endurance and retention characteristics of the memristor at **(A)** 25 °C and **(B)** 85 °C. HRS and LRS values were read at 0.2 V.

**Figure S6. NI-thickness-dependent switching variability and device-to-device statistics.
(A)** Representative I–V characteristics of Ag/TiO_2_/Au NI/Pt memristors with Au NI thicknesses of 0.5, 1.0, 1.5, and 2.0 nm. **(B)** Device-to-device statistics of HRS current measured at 0.2 V for ten devices per NI condition. **(C)** Device to device I-V characteristics of memristors with NI thickness of 1.5 nm. **(D)** Cumulative distribution function (CDF) from ten independent NI-1.5 devices over 100 cycles per device.

**Figure S7.** **(A)** Procedure used to estimate local electric-field amplification from the AFM height map. After plane removal, line flattening, and conversion to SI-calibrated height data, individual NI was segmented using an upper-percentile height threshold and a minimum-area criterion. For each NI, geometric descriptors including the effective radius r_eff_, peak height h_pk_, edge-average gradient (∣∇h∣), and local curvature (κ≃∇2h, under a smooth-surface approximation) were extracted. Under the adopted local-separation approximation, the normalized morphology-derived parameter was defined as β(x,y)=G/[G−h(x,y)], where β=1 corresponds to the planar reference condition. The same analysis was applied to all NI conditions for comparative evaluation. **(B)** Representative AFM topography image of the Au NI layer (1 µm × 1 µm field of view). **(C)** Heatmap of the morphology-derived β distribution. **(D)** Heatmap of the signed local curvature κ after plane removal and Gaussian smoothing (σ = 1 px), with zero-centered colors (red: κ>0, blue: κ<0 (nm^-1^)). The solid contours indicate β hotspots, defined as the top 10% of the field-enhancement distribution **(β ≥ 1.494)**. These hotspots are concentrated at two characteristic high-curvature regions, namely the NI summit and rim, and predominantly overlap with κ < 0 peak features. This spatial correspondence is consistent with geometrically prominent regions exhibiting stronger relative field concentration under the adopted approximation, without assigning individual filament-nucleation sites.

**Figure S8.** Trend of HRS variation as a function of reset amplitude.

**Figure S9.** **(A)** Schematic and corresponding breadboard implementation of the single shared capacitor-integration/sense-amplifier readout circuit used for paired-column comparison. The paired-column signals, I_L_ and I_R_, are integrated on matched 100 pF capacitors referenced to V_CM_ = 2.5V, buffered by LMC6001 voltage followers, differentially amplified by the INA128, and converted into the binary output Q by the LM311 comparator. **(B)** Photograph of the complete experimental setup with annotated signal flow. SET/RESET programming was performed using the parameter analyzer connected to the 32 × 32 ReRAM array in the probe station. The paired-column signals (I_L_, I_R_) were routed to the single shared readout circuit, and the hardware-generated comparator output Q was monitored using digital oscilloscope. **For long-bitstream acquisition, repeated SET–RESET–read cycles were performed at each fixed row/pair position, with a newly regenerated post-RESET HRS state preceding each recorded bit. After the prescribed number of bits was acquired at that position, the readout was sequentially moved to the next column pair. (C)** Twenty representative hardware-generated binary maps obtained from repeated readout operations of the memristor array. **(D)** Row-wise entropy statistics obtained from the repeated hardware acquisitions; error bars represent the standard deviation across the repeated measurements.

**Figure S10.** Effect of NI morphology on array-level entropy generation for NI = 1.0 nm **(A)** Resistance map and extracted bits from the memristor array. **(B)** Entropy distribution for each row, showing slightly larger spatial variation and residual bias than the optimized NI = 1.5nm.

**Figure S11.** Voltage-based FN representations of memristor with different temperature.

**Supplementary Note 1.** Monte Carlo stochastic sampling protocol using the memristor-derived bitstream.

To evaluate whether the statistically validated memristor-derived entropy stream can be used beyond random-bit generation, we implemented a software-level Monte Carlo probabilistic inference pipeline driven by the extracted bitstream. This protocol follows the broader concept that stochastic devices can serve as physical sampling resources for probabilistic neural computing.^[1]^

The entropy stream used in the Monte Carlo analysis was the hardware-generated binary stream directly acquired from the capacitor-integration/sense-amplifier output described in the main text and Figure S9. For each addressed row, paired HRS currents were integrated at the corresponding capacitive sense nodes and physically compared by the implemented sense amplifier, while the data-acquisition system was used to record the resulting binary outputs.

The resulting stream was used as the source of stochastic events in Monte Carlo inference. Specifically, during each Monte Carlo pass, binary sampling events were generated from the entropy stream and used to form a sampled weight configuration. The stochastic binary sampling process can be expressed as

b_ij_^(s)^=1[u_ij_^(s)^ <p_ij_],

where b_ij_^(s)^ is the sampled binary event for the i,j-th weight during the s-th Monte Carlo pass, p_ij_ is the sampling probability derived from the corresponding deterministic weight parameter, and u_ij_^(s)^  is a stochastic variable supplied by the entropy stream. The sampled binary event was then used to construct the stochastic weight state for each forward pass,

w_ij_^(s)^ = sign(w_ij_)⋅s_l_⋅b_ij_^(s)^,

sign(w_ij_) represents the sign of the trained deterministic weight and s_l_ is the layer-dependent scaling factor. Repeated stochastic forward passes produce an output ensemble,

{y^(1)^,y^(2)^,…,y^(S)^},

from which the predictive mean and predictive uncertainty are obtained. This ensemble-based estimation follows the standard Monte Carlo uncertainty-estimation principle in which multiple stochastic forward passes are aggregated to obtain a predictive distribution.^[2,3]^ The predictive mean and variance are calculated as

$$\bar{y}= \frac{1}{S}\sum_{s=1}^{S} y^{\left( s \right)}$$

and

$$\sigma_{y}^{2}=\frac{1}{S-1}\sum_{s=1}^{S} {(y^{(s)}-\bar{y})}^{2}$$

Here, S is the number of Monte Carlo stochastic forward passes. In this framework, the physical stream acts as a stochastic sampling primitive that drives the sampling events required for Monte Carlo probabilistic inference.

**Supplementary Note 2.** Interpretation of the FN-Derived Effective Transport-Gap Parameter

The parameter Δ_eff_ was obtained within the adopted Fowler–Nordheim (FN)-type phenomenological framework to provide a consistent description of cycle-dependent high-resistance-state (HRS) transport. In this framework, Δ_eff_ denotes the model-equivalent interfacial transport parameter required to reproduce the measured current under specified assumptions regarding the effective barrier height, local field-enhancement factor, and simplified barrier geometry. It should therefore not be interpreted as a direct measurement of the geometric separation between a residual Ag filament and the counter electrode.

Physically, the fitted Δ_eff_ may reflect the combined influence of several interfacial and filament-related factors, including the configuration of the residual Ag filament, the effective barrier height and profile, trap occupation, localized field concentration, and the number or spatial distribution of active conduction pathways. Because these contributions enter the FN-type response in a coupled manner, **the present electrical measurements do not uniquely resolve their individual contributions. The temperature-dependent measurements provide additional support for the increasing importance of FN-type field-driven transport in the high-field pre-SET regime, but they do not independently determine the individual microscopic quantities incorporated into Δ_eff_.** Accordingly, Δ_eff_ is treated as an effective descriptor of the overall interfacial transport state rather than as an independently determined structural quantity.

**For the cycle- and reset-condition comparisons discussed here,** the same barrier and field-scaling assumptions were applied throughout the analysis. Under this common parameterization, relative variations in Δ_eff_ provide a consistent basis for comparing the evolution of reset-dependent HRS transport states. In particular, changes in Δ_eff_ indicate that the effective transport condition has been modified, although they do not by themselves specify whether the modification originates predominantly from geometric separation, barrier reshaping, trap redistribution, field localization, or changes in the active transport pathways.

Because Δ_eff_ is mathematically inferred from the measured HRS current, the observed current–Δ_eff_ relationship represents an internal consistency of the adopted FN-type parameterization rather than an independent structural verification of physical nanogap evolution. The absolute values are therefore not presented as directly measured filament–electrode distances. Instead, Δ_eff_ is used to compare relative transport-state changes under identical modeling assumptions, together with the independently controlled reset-amplitude dependence and the measured HRS-current distributions.

**Table S1.** NIST SP 800-22 test results for the generated random bits.

| Test | p-value | Proportion | Result |
| --- | --- | --- | --- |
| Approximate Entropy | 0.423668904 | 109/110 | Pass |
| Block Frequency | 0.446340633 | 107/110 | Pass |
| Cumulative Sum (Backward) | 0.651251663 | 106/110 | Pass |
| Cumulative Sum (Forward) | 0.146362105 | 108/110 | Pass |
| FFT | 0.473522424 | 107/110 | Pass |
| Frequency | 0.486940243 | 110/110 | Pass |
| Longest Run (M=128) | 0.530018668 | 106/110 | Pass |
| Rank | 0.562252972 | 108/110 | Pass |
| Runs | 0.502934781 | 109/110 | Pass |

**Table S2. Benchmark comparison of representative memristor-based true random number generators using key device- and randomness-related metrics.**

| Device structure / type | Physical entropy source | Bit extraction | Scale | | Endurance | Bitstream | Post-processing |
| --- | --- | --- | --- | --- | --- | --- | --- |
| Pt/Ag/Ag:SiO_2_/Pt; diffusive^[6]^ | Stochastic Ag switching delay | Counter; 6 LSBs/event | Single device | | ≈ 10^7^ cycles | 76 Mbit (RT); 11 Mbit (85 °C) | None |
| Pt/HfO_2_/TiN; volatile TS^[7]^ | Stochastic delay/relaxation | Delay/relaxation-based circuit | Single-device circuit | | N.R. | N.R. | None |
| Pt/Ti/NbO_x_/Pt; Mott TS^[8]^ | Thermal-fluctuation-driven oscillation | Self-clocking oscillation parity | Single device | ≈ 4 × 10^8^ cycles | | 130 Mbit | None |
| Ag/h-BN/Ag; RTN^[9]^ | Charge trapping/detrapping RTN | Thresholded RTN → 19-bit NLFSR | Single device | N.R. | | 5.24 Mbit | 19-bit NLFSR conditioning |
| Ag/SiN_x_/n-Si; volatile TS^[10]^ | Stochastic CF switching delay | Dual-output NFSR | Paired device | N.R. | | 85 Mbit | NFSR extraction; no additional processing |
| Ag/TiO_2_/Au NI/Pt; ECM | Morphology-controlled field hotspots / reset-HRS dispersion | Paired-column current comparison | 32 × 32 array | 10^5^ cycles | | 12.0 Mbit | None |

**Table S3.** Comparison with recent stochastic memristive computing devices.

| Device structure | Device Type | Bit extraction scheme | Entropy-Source | Computing Application |
| --- | --- | --- | --- | --- |
| W/HfO_2_/Ti^[3]^ | Non-Volatile | Conductance-state sampling | Programmed conductance variability and device imperfections | Bayesian neural network with uncertainty quantification |
| Cu_0.1_Te_0.9_/HfO_2_/Pt ^[4]^ | Volatile | Voltage-controlled probabilistic switching / p-bit extraction | Stochastic threshold switching from diffusive Cu dynamics | p-bit-based probabilistic computing |
| Pt/Au/hBN/SiO_x_/Ag ^[5]^ | Volatile | Comparator-based stochastic number encoding from switching events | Stochastic Ag-ion diffusion and filament formation/rupture | Stochastic computing for error-tolerant edge detection |
| Ag/TiO_2_/Au NI /Pt | Non-Volatile | Morphology-defined field localization and reset-modulated nanogap landscape | local field hotspots and reset-induced nanogap variability | Monte Carlo probabilistic sampling |
|  |  |  |  |  |

**Table S4.** Comparison with representative HRS-variability-based filamentary ReRAM TRNGs

| Study | Entropy source | Main control | Bit extraction | Design-level contribution |
| --- | --- | --- | --- | --- |
| Yang et al.^[11]^ | Stochastic RESET-pulse count | Pulse amplitude and width | Counter bits | Electrical programming of a fixed RRAM |
| Akbari et al.^[12]^ | Cycle-to-cycle HRS variability | Cycle separation and device comparison | HRS-to-delay comparison | HRS variability with circuit-level conditioning |
| Arumí et al.^[13]^ | Cycle-to-cycle HRS variability | HRS-dependent oscillator bias | CSRO frequency sampling | Circuit conversion of measured HRS variability |
| This work | Morphology-modulated HRS landscape | Au NI thickness under a common reset/read protocol | Paired-column comparison in a 32 × 32 array | Materials-level programming of the entropy–operability landscape |

**References**

1. S. Misra, L. C. Bland, S. G. Cardwell, J. A. C. Incorvia, C. D. James, A. D. Kent, C. D. Schuman, J. D. Smith, J. B. Aimone, Adv. Mater. 2023, 35, 2204569.
2. Y. Gal, Z. Ghahramani, Proc. 33rd Int. Conf. Mach. Learn. 2016, 48, 1050.
3. D. Bonnet, T. Hirtzlin, A. Majumdar, T. Dalgaty, E. Esmanhotto, V. Meli, N. Castellani, S. Martin, J.-F. Nodin, G. Bourgeois, J.-M. Portal, D. Querlioz, E. Vianello, Nat. Commun. 2023, 14, 7530.
4. K. S. Woo, J. Kim, J. Han, W. Kim, Y. H. Jang, C. S. Hwang, Nat. Commun. 2022, 13, 5762.
5. L. Song, P. Liu, J. Pei, Y. Liu, S. Liu, S. Wang, L. W. T. Ng, T. Hasan, K.-P. Pun, S. Gao, G. Hu, Nat. Commun. 2025, 16, 4550.
6. H. Jiang, D. Belkin, S. E. Savel’ev, S. Lin, Z. Wang, Y. Li, S. Joshi, R. Midya, C. Li, M. Rao, M. Barnell, Q. Wu, J. J. Yang, Q. Xia, Nat. Commun. 2017, 8, 882.
7. K. S. Woo, Y. Wang, J. Kim, Y. Kim, Y. J. Kwon, J. H. Yoon, W. Kim, C. S. Hwang, Adv. Electron. Mater. 2019, 5, 1800543.
8. G. Kim, J. H. In, Y. S. Kim, H. Rhee, W. Park, H. Song, J. Park, K. M. Kim, Nat. Commun. 2021, 12, 2906.
9. S. Pazos, W. Zheng, T. Zanotti, F. Aguirre, T. Becker, Y. Shen, K. Zhu, Y. Yuan, G. Wirth, F. M. Puglisi, J. B. Roldán, F. Palumbo, M. Lanza, Nanoscale 2023, 15, 2171.
10. X. Yan, Z. Zhang, Z. Guan, Z. Fang, Y. Zhang, J. Zhao, J. Sun, X. Han, J. Niu, L. Wang, X. Jia, Y. Shao, Z. Zhao, Z. Guo, B. Bai, Front. Phys. 2024, 19, 13202.
11. B. Yang, D. Arumí, S. Manich, et al., Electronics 2021, 10, 1831.
12. M. Akbari, S. Mirzakuchaki, D. Arumí, et al., IEEE Access 2023, 11, 66682–66693.
13. D. Arumí, S. Manich, A. Gómez-Pau, et al., IEEE J. Explor. Solid-State Comput. Devices Circuits 2023, 9, 92–98.
